# Thermoresponsive Injectable Self-Healing Hydrogel Loaded with Self-Regenerating Photothermal Agent for Synergistic Photothermal–Thermodynamic–Chemodynamic Therapy for Pancreatic Cancer

**DOI:** 10.3390/polym18131620

**Published:** 2026-06-29

**Authors:** Junhang Li, Weizhong Yuan

**Affiliations:** School of Materials Science and Engineering, Tongji University, Shanghai 201804, China; 2331466@tongji.edu.cn

**Keywords:** thermoresponsive hydrogel, injectable self-healing hydrogel, photothermal therapy, thermodynamic therapy, pancreatic cancer

## Abstract

Pancreatic ductal adenocarcinoma is highly malignant with poor prognosis. Its dense tumor microenvironment severely limits the efficacy of conventional chemotherapy and causes severe side-effects. Herein, we adopt the established Schiff-base crosslinked thermoresponsive injectable self-healing poly(2-(2-methoxyethoxy)ethyl methacrylate-*co*-oligo(ethylene glycol) methyl ether methacrylate-*co*-aldehyde 2-hydroxyethyl methacrylate)/carboxymethyl chitosan (APMOH/CMCS) hydrogel as the delivery scaffold. By regulating monomer composition, the volume phase transition temperature (T_VPT_) of the hydrogel was tuned to around 43 °C to match the therapeutic temperature requirement. Subsequently, copper–metal organic framework (Cu-MOF) nanoparticles co-loaded with 2,2′-azobis(2-methylimidazoline) dihydrochloride (AIPH) and 2,2′-azinobis(3-ethylbenzothiazoline-6-sulfonic acid) cationic radicals (ABTS·^+^) (denoted as AB@Cu-MOF) were uniformly incorporated into the hydrogel network. Under near-infrared (NIR) irradiation, ABTS·^+^ acts as a photothermal agent to generate hyperthermia for tumor ablation; the elevated temperature further activates AIPH to produce alkyl radicals, which can oxidize inactivated ABTS back to ABTS·^+^ and construct a sustainable photothermal therapy–thermodynamic therapy (PTT-TDT) circulation. Meanwhile, Cu-MOF can consume intracellular glutathione (GSH) to protect active components from deactivation and initiate chemodynamic therapy (CDT) via Fenton-like reactions to produce toxic reactive oxygen species. Benefiting from the thermoresponsive characteristic, the hydrogel undergoes volume shrinkage upon heating, achieving NIR-triggered on-demand drug release with a cumulative release rate of 81.1%. In vitro and in vivo experiments verified that this integrated platform realizes remarkable triple synergistic efficacy of PTT, TDT, and CDT. The tumor volume of the treatment group was merely 13.3% of the control group, and the system also exhibited excellent biocompatibility. Collectively, it offers a feasible and promising intelligent platform for precise local treatment of pancreatic cancer.

## 1. Introduction

Pancreatic cancer remains one of the most lethal malignant tumors worldwide [1,2]. Among its subtypes, pancreatic ductal adenocarcinoma (PDAC) accounts for most cases and presents an extremely poor prognosis, with a 5-year survival rate of merely approximately 11% [3,4]. Surgical resection is currently the only potential radical treatment approach. Nevertheless, 80–85% of patients are diagnosed at advanced stages and thus miss the optimal surgical opportunity [5,6,7,8]. In the field of chemotherapy, although the folfirinox regimen and gemcitabine/albumin-bound paclitaxel have been established as standard first-line palliative treatment protocols, the unique pathological characteristics of PDAC severely restrict therapeutic outcomes [9,10,11,12]. Up to 80% of tumor volume is occupied by stromal matrix, forming a dense physical barrier [13,14,15,16]. Additionally, most pancreatic tumors suffer from disordered blood perfusion, which hinders the penetration of chemotherapeutic drugs into tumor lesions and ultimately leads to poor tumor responsiveness to most chemotherapeutic agents [10,16,17,18]. Furthermore, PDAC is featured with a highly immunosuppressive tumor microenvironment accompanied by hypoxia and excessive glutathione (GSH), which further aggravates therapeutic resistance and limits the clinical efficacy of emerging strategies such as immunotherapy [19,20,21,22,23,24].

In recent years, nanotechnology has attracted increasing attention in cancer diagnosis and treatment [25,26,27]. Nanomaterials accumulate in tumor tissues relying on the enhanced permeability and retention effect (EPR), thereby improving therapeutic efficacy [28,29,30]. Recently, nanomaterial-based therapeutic modalities mainly include photothermal therapy (PTT), sonodynamic therapy (SDT), chemodynamic therapy (CDT), and thermodynamic therapy (TDT) [31,32,33,34,35,36]. PTT and TDT possess inherent synergistic potential [37]. Under near-infrared light (NIR) irradiation, photothermal agents rapidly convert light energy into thermal energy via PTT. When the temperature rises above 43 °C, it can induce protein denaturation and apoptosis of tumor cells [38]. TDT eliminates tumor cells through oxygen-independent free radicals such as alkyl radicals, which can be generated by the heat-sensitive molecule 2,2′-azobis(2-methylimidazoline) dihydrochloride (AIPH) under heating conditions in an oxygen-deficient environment [39,40,41,42]. More importantly, the hyperthermia effect derived from PTT can not only ablate tumor tissues directly but also serve as a heat source to trigger the decomposition of AIPH, laying a solid synergistic foundation for combined PTT-TDT treatment and overcoming the insufficient therapeutic effect of monotherapy [36]. CDT relies on Fenton/Fenton-like reactions between transition-metal-based materials and endogenous hydrogen peroxide (H_2_O_2_) within the tumor microenvironment. Cytotoxic hydroxyl radicals (·OH) are generated in situ to eliminate cancer cells, and this therapy exhibits universal therapeutic efficacy against hypoxic tumors.

Nevertheless, existing combinatorial therapeutic platforms still suffer from critical drawbacks. The dense stromal matrix and disordered vasculature characteristic of PDAC severely hinder the intratumoral accumulation of systemically administered chemotherapeutics [43,44,45]. In this context, injectable thermosensitive hydrogels have been developed as attractive localized delivery scaffolds [46,47,48]. Intratumoral administration circumvents the stromal barrier and prevents systemic drug dilution and clearance, while the inherent thermoresponsiveness of such hydrogels permits NIR-activated controlled drug release to mitigate off-target toxicity to healthy tissues [49,50,51]. Unfortunately, most reported hydrogel matrices act only as passive cargo carriers rather than participators in synergistic therapeutic cascades and thus cannot address bottlenecks such as photothermal agent deactivation and inadequate therapeutic synergy [52,53].

At present, numerous composite hydrogel/nano-systems integrated with photothermal agents, metal–organic frameworks, and reactive oxygen species generating systems have been applied for tumor therapy. Among them, Cu-MOF is a commonly used functional material in CDT-based therapeutic systems. Cupric ions (Cu^2+^) can consume excess GSH and generate cuprous ions (Cu^+^). The resultant Cu^+^ triggers Fenton-like reactions to produce cytotoxic ·OH and mediate the CDT effect [54]. Nevertheless, Cu-MOF can only partially deplete GSH. Tumor cells continuously synthesize new GSH to rebuild the antioxidant system, and the regenerated GSH will scavenge hydroxyl radicals persistently and impair the efficacy of CDT [55,56]. In addition, most systems constructed based on the inherent synergistic effects of PTT and TDT adopt inorganic metallic nanoparticles as photothermal agents to drive the decomposition of thermodynamic agent AIPH while ignoring the cytotoxicity of such inorganic photothermal materials [57,58]. In contrast, small-molecule photothermal agents possess superior biodegradability, lower cytotoxicity, and better loading performance within hydrogel carriers, showing remarkable advantages in biocompatibility, clinical transformation potential, and compatibility for combined therapy [59,60,61,62]. However, small-molecule photothermal agents are susceptible to reduction and inactivation by GSH, which cuts off the heat source for thermodynamic reactions and eventually terminates the entire therapeutic cycle. Furthermore, most existing systems simply physically mix different functional components, and no interactive cascaded reactions are established among individual functional units.

To address the above limitations, a novel PTT-TDT-CDT composite system was designed in this work. Cu-MOF co-loaded with photothermal agent 2,2′-azinobis(3-ethylbenzothiazoline-6-sulfonic acid) cationic radicals (ABTS·^+^) and thermodynamic agent AIPH was selected as the core functional component and uniformly dispersed into injectable thermoresponsive hydrogels. The hydrogel was composed of poly(2-(2-methoxyethoxy)ethyl methacrylate-*co*-oligo(ethylene glycol) methyl ether methacrylate-*co*-aldehyde 2-hydroxyethyl methacrylate ((P(MEO_2_MA-*co*-OEGMA-*co*-HEMA-CHO), APMOH)) and carboxymethyl chitosan (CMCS), which formed dynamic crosslinking via Schiff base bonds between aldehyde and amino groups on molecular chains. This system retains the inherent capabilities of Cu-MOF to deplete GSH and mediate CDT and innovatively introduces an ABTS·^+^ self-regeneration mechanism to construct stable PTT-TDT circulation. The pre-consumption of GSH by Cu-MOF effectively prevents the premature inactivation of ABTS·^+^ and ensures the smooth initiation of PTT-TDT circulation. Under near-infrared irradiation, ABTS·^+^ converts light energy into thermal energy. On one hand, tumor ablation is realized via photothermal effect. On the other hand, the elevated temperature induces the decomposition of AIPH to generate alkyl radicals and activate TDT for tumor suppression. Affected by the high concentration of GSH in the tumor microenvironment, ABTS·^+^ is easily reduced into photoinactive ABTS. The alkyl radicals derived from AIPH can re-oxidize inactive ABTS back into active ABTS·^+^, realizing the self-regeneration of photothermal agents and establishing sustainable PTT-TDT self-circulation. Meanwhile, when the temperature rises to reach the volume phase transition temperature (T_VPT_) of the hydrogel upon near-infrared irradiation, the material undergoes hydrophilic–hydrophobic phase transition and volume contraction, achieving precise and controlled release of functional components.

**Scheme 1:**
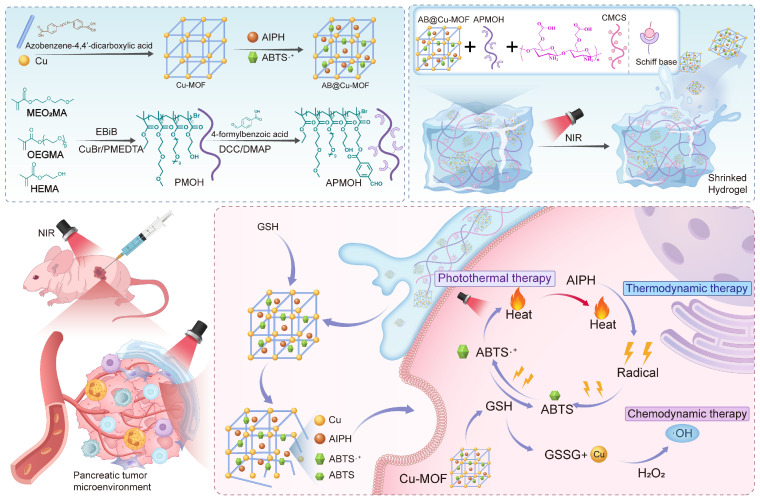
Preparation of APMOH/CMCS thermoresponsive injectable hydrogel loaded with AB@Cu-MOF and PTT-TDT-CDT synergistic therapy for pancreatic cancer.

Overall, this design possesses multiple advantages over conventional systems. Alkyl radicals generated from TDT compensate for the deficiency caused by scavenged hydroxyl radicals in CDT. The adoption of small-molecule ABTS·^+^ avoids the cytotoxicity of inorganic materials, and the proposed self-regeneration strategy solves the problem of irreversible inactivation of small-molecule photothermal agents. Moreover, the heat produced by PTT serves three key processes simultaneously, tumor thermal ablation, thermal decomposition of AIPH, and stimuli-responsive drug release of thermoresponsive hydrogels, enabling high synergism among all functional modules. Ultimately, this integrated system achieves mutually reinforcing and synergistic effects of PTT, TDT, and CDT, offering a promising new strategy for the precise treatment of pancreatic cancer (see Scheme 1).

## 2. Materials and Methods

### 2.1. Materials

Polyvinylpyrrolidone (PVP, *M*_w_ = 8000–12,000 g/mol, 95.0%), N,N-dimethylformamide (DMF, 99.8%), azobenzene-4,4′-dicarboxylic acid (98.0%), 2,2′-azino-bis(3-ethylbenzothiazoline-6-sulfonic acid) diammonium salt (ABTS, 98.0%), 2,2′-azobis(2-methylimidazoline) dihydrochloride (AIPH, 98%), potassium persulfate (K_2_S_2_O_8_, 99.5%), ethyl 2-bromoisobutyrate (EBiB, 98.0%), copper(I) bromide (CuBr, 99.0%), N,N,N′,N′,N″-pentamethyldiethylenetriamine (PMDETA, 98.0%), 2-(2-methoxyethoxy)ethyl methacrylate (MEO_2_MA, 97.0%), oligo(ethylene glycol) methyl ether methacrylate (OEGMA, *M*_n_ = 475 g/mol), 2-hydroxyethyl methacrylate (HEMA, 99.0%), 4-formylbenzoic acid (98.0%), N,N′-dicyclohexylcarbodiimide (DCC, 99.0%), and 4-dimethylaminopyridine (DMAP, 99.0%) were all purchased from Adamas Reagent Co., Ltd., Shanghai, China. Glacial acetic acid (99.8%), basic alumina (200–300 mesh, FCP for chromatography), and neutral alumina (200–300 mesh, FCP for chromatography) were obtained from Sinopharm Chemical Reagent Co., Ltd., Shanghai, China. Anhydrous ethanol (99.7%), copper(II) chloride dihydrate (CuCl_2_·2H_2_O, 99.0%), and tetrahydrofuran (THF, 99.5%) were supplied by Greagent, Shanghai, China. Dulbecco’s modified eagle medium (DMEM), newborn calf serum (NBCS) and fetal bovine serum (FBS) were supplied by Gibco, Grand Island, NY, USA. KeyGEN BioTECH supplied the Cell Counting Kit-8 (CCK-8) assay kit, calcein-acetoxy methyl/propidium iodide (Calcein-AM/PI) staining kit, and 2′,7′-dichlorodihydrofluorescein diacetate (DCFH-DA), ROSup (reactive oxygen species positive control reagent), Nanjing, China.

CuBr was repeatedly soaked, stirred, and washed with glacial acetic acid until the supernatant became nearly colorless and transparent. Subsequently, it was rinsed several times with anhydrous ethanol, vacuum-dried at room temperature, and stored in a sealed container away from light. The monomers of MEO_2_MA, OEGMA, and HEMA were purified through column chromatography over basic alumina to remove polymerization inhibitors. Both basic alumina and neutral alumina were activated in an oven at 120 °C for 2 h in advance. Unless otherwise specified, all other reagents and raw materials were commercially available analytical grade materials and could be used directly without further purification.

### 2.2. Preparation of Cu-MOF

Cu-MOF was prepared and modified via a one-step solvothermal reduction method, and the detailed procedures are described as follows [63]. Firstly, 1 g of PVP was added into the mixed solvent consisting of 20 mL of ethanol and 20 mL of DMF, followed by ultrasonic treatment until complete dissolution. Meanwhile, 459 mg of CuCl_2_·2H_2_O and 108 mg of azobenzene-4,4′-dicarboxylic acid were dissolved in 20 mL of DMF. The two above-prepared solutions were blended and subjected to continuous ultrasonication for 30 min and then transferred into an oven to react at 100 °C for 13 h. After the reaction finished, the obtained Cu-MOF was collected through centrifugation and repeatedly washed with DMF, ethanol, and deionized water in sequence. Finally, the dried Cu-MOF powder was obtained after vacuum drying at 60 °C for 24 h.

### 2.3. Preparation of AB@Cu-MOF

ABTS·^+^ radical cations were prepared via oxidation of ABTS with K_2_S_2_O_8_ [64]. Typically, ABTS solution (10 mg/mL) was mixed evenly with K_2_S_2_O_8_ solution (1.78 mg/mL) at a volume ratio of 1:1. The mixed solution was incubated away from light for 12 h and stored in dark conditions for subsequent use. The dried Cu-MOF powder was dispersed in deionized water to prepare an aqueous solution with a concentration of 300 μg/mL. Afterwards, ABTS·^+^ solution and AIPH powder were added successively to adjust their final concentrations to 600 μg/mL, respectively. The resultant mixture was stirred vigorously for 24 h. After centrifugation and repeated washing with deionized water, the solid product was dried to obtain AIPH and ABTS·^+^ co-loaded Cu-MOF powder, termed as AB@Cu-MOF. To evaluate the loading performance of Cu-MOF, the encapsulation efficiency (EE) and loading efficiency (LE) of AIPH and ABTS·^+^ were determined. A 3 mL mixed solution was prepared containing Cu-MOF at 300 μg/mL, AIPH at 600 μg/mL, and ABTS·^+^ at 600 μg/mL. After stirring for 24 h, the mixture was centrifuged at 12,000 rpm. The absorbance of the supernatant was determined through ultraviolet-visible (UV-Vis) spectrophotometry at 300 nm for AIPH and 734 nm for ABTS·^+^. The mass of free substances *m*_free_ was further calculated based on the established calibration curves and the volume of the supernatant. The total feeding mass (*m*_total_) of both AIPH and ABTS·^+^ was 1800 μg. The mass of loaded substances was calculated as *m*_loaded_ = *m*_total_ − *m*_free_. EE and LE were calculated according to Equations (1) and (2), respectively:

(1) $$\mathrm{EE}\;(\%)\;=\;\frac{m_{loaded}}{m_{total}}\;\times \;100\%$$

(2) $$\mathrm{LE}\;(\%)=\frac{m_{loaded}}{m_{AB@Cu\text{-}MOF}}\;\times \;100\%$$

where $m_{AB@Cu\text{-}MOF}$ is the total mass of dried AB@Cu-MOF powder.

For control experiments, Cu-MOF samples loaded with single AIPH or single ABTS·^+^ were also fabricated. Specifically, only AIPH or ABTS·^+^ was added into 300 μg/mL of Cu-MOF aqueous solution to reach a final concentration of 600 μg/mL, and all other preparation procedures were consistent with those for AB@Cu-MOF. The single AIPH-loaded and single ABTS·^+^-loaded samples were termed as A@Cu-MOF and B@Cu-MOF, respectively.

### 2.4. Synthesis of Thermoresponsive P(MEO_2_MA-co-OEGMA-co-HEMA) (PMOH) Copolymer

Linear random copolymer PMOH was synthesized via atom transfer radical polymerization (ATRP) using EBiB as the initiator and CuBr/PMDETA as the catalytic system, with MEO_2_MA, OEGMA, and HEMA as monomers. The detailed procedures are shown as follows. EBiB (0.166 g, 0.85 mmol), MEO_2_MA (10.84 g, 57.59 mmol), OEGMA (6.08 g, 12.79 mmol), and HEMA (1.11 g, 8.53 mmol) were dissolved in 20 mL of anhydrous DMF and stirred for 15 min. Subsequently, CuBr (0.122 g, 0.85 mmol) and PMDETA (178.46 μL, 0.85 mmol) were added into the above mixture. After repeated vacuumizing and nitrogen purging to remove dissolved oxygen, the mixture was continuously stirred and reacted at 70 °C for 12 h. Upon completion of the reaction, the crude product was passed through a neutral alumina column to remove copper-based catalysts, followed by dialysis in deionized water for 72 h with a molecular weight cut-off of 3500 Da. The purified PMOH copolymer was finally obtained after freeze-drying.

^1^H NMR (DMSO-*d*_6_, *δ*, ppm): 4.76 (-O*H*), 4.02 (-COO-C*H*_2_-), 3.90 (-C*H*_2_-OH), 3.52 (-O-C*H*_2_-CH_2_-O-), 3.18 (-O-C*H*_3_), 1.74 (-C-C*H*_2_-C-), 1.16 (-CH_2_-C*H*_3_), 0.80 (-C-C*H*_3_-).

### 2.5. Synthesis of Thermoresponsive APMOH Copolymer

PMOH (4.978 g, 0.61 mmol) was dissolved in dried THF (30 mL). Subsequently, 4-formylbenzoic acid (0.5 g, 3.33 mmol), DCC (1 g, 4.85 mmol), and DMAP (30 mg, 0.25 mmol) were added into the solution. The system was purged with nitrogen after vacuumization and stirred at 25 °C for 20 h. After the reaction was completed, the mixture was subjected to suction filtration. The resulting filtrate was dialyzed against deionized water for 72 h using dialysis bags with a 3500 Da molecular weight cut-off, and pale yellow APMOH copolymer was ultimately harvested after lyophilization.

^1^H NMR (DMSO-*d*_6_, *δ*, ppm): 10.14 (-C*H*O), 8.18 (-C_6_*H*_6_-), 4.01 (-CH_2_-C*H*_2_-COO-), 3.51 (-O-C*H*_2_-C*H*_2_-O-), 3.34 (-O-C*H*_3_), 1.77 (-C-C*H*_2_-C-), 1.21 (-CH_2_-C*H*_3_), 0.80 (-C-C*H*_3_-).

### 2.6. Preparation of Thermoresponsive APMOH/CMCS Self-Healing Nanocomposite Hydrogel

APMOH solution (3 wt%) was mixed with CMCS solution (10 wt%) at a volume ratio of 1:1. After thorough stirring, the mixture was kept static at room temperature for 10 min to form APMOH/CMCS self-healing hydrogel, which was denoted as H. AB@Cu-MOF (2 mg/mL) was uniformly dispersed into APMOH solution via ultrasonic treatment, followed by blending with CMCS solution to prepare homogeneous nanocomposite hydrogel termed as H + AB@Cu-MOF.

In addition, APMOH/CMCS hydrogel containing 2 mg/mL of Cu-MOF was termed as H + Cu-MOF, that containing 2 mg/mL of A@Cu-MOF was defined as H + A@Cu-MOF, and that containing 2 mg/mL of B@Cu-MOF was termed as H + B@Cu-MOF. The APMOH/CMCS hydrogel simultaneously containing AIPH and ABTS·^+^ both at the concentration of 2 mg/mL was designated as H + AB.

### 2.7. Characterization

The microscopic morphologies of Cu-MOF and AB@Cu-MOF were characterized using a JEOL JEM-2100F transmission electron microscope (TEM, JEOL Ltd., Tokyo, Japan). The hydrodynamic radius (*R*_h_) of Cu-MOF and AB@Cu-MOF was measured and analyzed with a Malvern Zetasizer Nano ZS90 dynamic light scattering instrument (DLS, Malvern Panalytical, Malvern, UK). Elemental valence state analysis of samples was carried out using an ESCALAB 250Xi X-ray photoelectron spectrometer (XPS, Thermo Fisher Scientific, Waltham, MA, USA). Phase analysis of Cu-MOF samples was performed on a Rigaku D/max 2550VB3+/PC X-ray diffractometer (XRD, Rigaku Corporation, Tokyo, Japan). The absorbance of samples was determined via a METASH UV-9000S double-beam ultraviolet-visible spectrophotometer (UV-Vis, Shanghai Metash Instruments Co., Ltd., Shanghai, China). The three-dimensional surface network structure of freeze-dried hydrogels was observed using a Nova Nano SEM 450 scanning electron microscope (SEM, Thermo Fisher Scientific, Waltham, MA, USA). The structures of solid PMOH, solid APMOH, and freeze-dried APMOH/CMCS hydrogel were characterized using a Thermo Scientific Nicolet iS20 Fourier transform infrared spectrometer (FT-IR, Thermo Fisher Scientific, Waltham, MA, USA). The chemical structure and segment composition of PMOH and APMOH copolymers were analyzed using a Bruker 400 MHz nuclear magnetic resonance spectrometer (^1^H NMR, Bruker Corporation, Karlsruhe, Germany) with deuterated dimethyl sulfoxide (DMSO-*d*_6_) as the solvent. Molecular weight and molecular weight distributions of PMOH and APMOH were determined using an Agilent gel permeation chromatography (GPC, Agilent Technologies, Santa Clara, CA, USA) system equipped with a refractive index detector (RID), using tetrahydrofuran (THF) as the mobile phase.

### 2.8. Thermoresponsiveness of Copolymers and Hydrogels

The transmittance variations of PMOH aqueous solution, APMOH aqueous solution, and APMOH/CMCS hydrogel at different temperatures were measured by using a U-3310 ultraviolet-visible spectrophotometer. The test temperature range was set from 25 °C to 80 °C, and each temperature point was kept for 5 min with the detection wavelength ranging from 400 nm to 700 nm. The transmittance of the system remains high below the T_VPT_, while drastic turbidity and sharp transmittance reduction occur once the temperature exceeds T_VPT_. The T_VPT_ of each sample was calculated as the average value of the onset and offset temperatures of the transmittance transition region.

### 2.9. Rheological Test

Rheological measurements of hydrogels were performed on a Thermo Fisher HAAKE MARS rheometer equipped with a 20 mm parallel plate fixture at a fixed temperature of 25 °C. (1) The storage modulus (G′) and loss modulus (G″) of APMOH/CMCS hydrogel were tested via strain sweep ranging from γ = 0.01% to 3500% at a constant angular frequency of 1 rad/s. (2) The self-healing property of APMOH/CMCS hydrogel was evaluated through alternate strain sweep at 1 rad/s. The oscillatory strain was switched from low strain to high strain of 1000% with an interval of 60 s for each strain period.

### 2.10. Degradation Test of Hydrogels

APMOH/CMCS hydrogels were first freeze-dried for 12 h, and the initial dry weight was recorded as (W1). Subsequently, the samples were immersed in 2 mL of PBS solution at pH 5.8 and placed in an oven at 37 °C to simulate the tumor microenvironment for a 7-day in vitro degradation experiment. The dry weight (W2) of freeze-dried samples was recorded every day. The in vitro degradation degree of hydrogels was characterized by mass residual rate, which was calculated according to Equation (3).

(3) $$\mathrm{Mass}\;\mathrm{Remaining}=\frac{W2}{W1}\;\times \;100\%$$

### 2.11. Self-Regeneration Performance of Photothermal Agent in AB@Cu-MOF

To verify that glutathione can consume photothermal agent ABTS·^+^ within AB@Cu-MOF and that ABTS·^+^ can be regenerated under NIR light irradiation, relevant experiments were carried out as follows. AB@Cu-MOF and GSH were added into PBS solution to achieve final concentrations of 300 μg/mL and 0.5 mg/mL, respectively. The mixture was pre-treated in an oven at 37 °C for 30 min to simulate the effect of excessive GSH on AB@Cu-MOF in the tumor microenvironment. The changes in UV absorbance of AB@Cu-MOF before and after GSH treatment were detected using a ultraviolet-visible spectrophotometer. After pretreatment, the solution was irradiated with an 808 nm NIR laser emitter (Shanghai Hengjin Electronics Co., Ltd., Shanghai, China) at a power density of 1 W/cm^2^. The absorbance changes in the range of 450–950 nm were recorded every 2 min under NIR irradiation. Because ABTS·^+^ exhibits a characteristic absorption peak at 734 nm, its regeneration behavior can be evaluated according to the absorbance variation at this wavelength. In addition, L-ascorbic acid was introduced to conduct alkyl radical scavenging experiments for verifying the dominant role of alkyl radicals in activating ABTS·^+^. After AB@Cu-MOF was pretreated with GSH for 30 min, L-ascorbic acid was added to the system at final concentrations of 0.2 mg/mL and 0.8 mg/mL. At the above concentrations, L-ascorbic acid exhibits excellent solubility and shows no UV absorption at 734 nm, which eliminates spectral interference during detection. Under the unified NIR irradiation conditions, the absorbance at 734 nm was measured every 2 min to clarify the correlation between alkyl radicals and the activation of ABTS·^+^.

### 2.12. Photothermal Property Measurement and Thermal Imaging of Nanocomposite Hydrogels

To confirm the enhanced photothermal effect derived from the self-regeneration of photothermal agents in AB@Cu-MOF, the temperature elevation curves of H_2_O, H + B@Cu-MOF, H + AB, and H + AB@Cu-MOF were compared under NIR irradiation at 808 nm with identical power density of 1 W/cm^2^. Before being incorporated into hydrogels, the nanomaterials in groups of H + B@Cu-MOF, H + AB, and H + AB@Cu-MOF were pre-incubated with 0.5 mg/mL of GSH at 37 °C for 30 min to simulate the influence of intratumoral GSH on photothermal agent ABTS·^+^. The temperature variation (ΔT) was recorded every 30 s via an infrared thermal imaging camera. To verify the photothermal cycling stability of the samples and the superior self-regenerative photothermal performance of AB@Cu-MOF, three heating–cooling cycles of H + B@Cu-MOF, H + AB, and H + AB@Cu-MOF were conducted. Specifically, each composite hydrogel was irradiated with NIR light for 5 min followed by natural cooling for another 5 min, and the corresponding ΔT was recorded every 30 s using an infrared thermal imaging camera.

### 2.13. Photothermally Enhanced Thermodynamic Performance

The enhanced photothermal effect of the AB@Cu-MOF system originates from the regeneration of ABTS·^+^, which is probably driven by the continuous generation of alkyl radicals to oxidize GSH-reduced ABTS·^+^. To verify this hypothesis, a DCFH-DA probe was used to detect the production level of alkyl radicals in the system. DCFH-DA was dissolved in DMSO to prepare 1 mM of stock solution, which was further diluted to 10 μM of working solution with PBS before experiment. The solution was incubated at 37 °C away from light for 30 min to be hydrolyzed into non-fluorescent DCFH. Under dark conditions, DCFH working solution was mixed with different samples, including H_2_O, Cu-MOF, A@Cu-MOF, and AB@Cu-MOF. Among them, Cu-MOF, A@Cu-MOF, and AB@Cu-MOF were all dispersed in PBS at a concentration of 300 μg/mL and pre-treated with 0.5 mg/mL of GSH solution at 37 °C for 30 min in advance. Afterwards, the mixtures were irradiated with 808 nm NIR light at 1 W/cm^2^ for 12 min, and samples were collected every 2 min. Fluorescence intensity was measured using a fluorescence spectrophotometer with an excitation wavelength of 488 nm and an emission wavelength of 525 nm. The cumulative production of alkyl radicals was characterized by the variation of fluorescence intensity (F_t_ − F_0_).

### 2.14. Glutathione Peroxidase (GPx)-like Catalytic Activity of Cu-MOF

High-concentration GSH in the tumor microenvironment tends to reduce and consume ABTS·^+^ in AB@Cu-MOF in advance, thereby failing to effectively initiate the NIR-triggered PTT-TDT cycle. As the main carrier for loading AIPH and ABTS·^+^, Cu-MOF can consume partial GSH in advance to prevent rapid reduction of ABTS·^+^. To confirm this hypothesis, DTNB was adopted as a specific probe for GSH. It can react with GSH to generate TNB- with a strong characteristic absorption peak at 412 nm, and the GSH content can be quantified according to absorbance changes. PBS dispersion of Cu-MOF at a final concentration of 300 μg/mL was mixed with 0.5 mg/mL of GSH and 0.039 mg/mL of DTNB and incubated at 37 °C for 40 min. The UV-vis absorption spectra ranging from 220 nm to 550 nm were recorded every 5 min. Meanwhile, Cu-MOF with concentrations ranging from 100 to 500 μg/mL was incubated with GSH for 30 min to detect absorption variations to explore the concentration-dependent effect on GSH consumption.

### 2.15. Peroxidase (POD)-like Catalytic Activity of AB@Cu-MOF

Cu-MOF can be reduced to generate Cu^+^ after consuming GSH. To verify the Fenton-like catalytic performance of Cu^+^, methylene blue (MB) was used as a probe to detect ·OH production. Hydroxyl radicals can oxidize and destroy the conjugated structure of MB, leading to a decrease in its characteristic absorption intensity at 664 nm. Firstly, PBS dispersion of AB@Cu-MOF at 300 μg/mL was incubated with 0.5 mg/mL GSH at 37 °C for 30 min to fully produce Cu^+^ via reduction. Subsequently, 200 μL of pretreated AB@Cu-MOF solution, 200 μL of H_2_O_2_ solution (3–6%), and 3 μL of MB solution (2 mg/mL) were mixed and incubated continuously at 37 °C for 50 min. The UV-vis absorbance at 664 nm was detected every 10 min. Meanwhile, AB@Cu-MOF with concentrations ranging from 100 to 500 μg/mL was set up to investigate the concentration dependence of its POD-like catalytic activity.

### 2.16. Controlled Release Behavior of Nanocomposite Hydrogels

APMOH/CMCS hydrogel possesses temperature responsiveness. When the temperature exceeds its T_VPT_, structural collapse and volume shrinkage occur, which accelerates the release of encapsulated substances. To verify the stimuli-responsive release behavior, 1 mL of APMOH/CMCS hydrogel containing 2 mg/mL of AB@Cu-MOF was sealed in a dialysis bag with a molecular weight cut-off of 3500 Da. The dialysis bag was immersed in 25 mL of PBS solution at pH 5.8 to simulate the pH condition of the tumor microenvironment. One group was incubated in a constant-temperature shaking incubator at 37 °C, and the other group was subjected to pulsed NIR irradiation (808 nm, 1 W/cm^2^). Within the initial 10 h, 1 mL of release medium was collected every hour, and the absorbance at 800 nm was measured using a UV-vis spectrophotometer to quantify the released AB@Cu-MOF, followed by supplementing 1 mL of fresh PBS. The sampling interval was prolonged in the subsequent period to explore the effect of temperature on controlled release. The cumulative release rate was calculated according to Equation (4).

(4) $$E\;(\%)=\frac{C_{n}V_{0}+V\sum_{1}^{n-1}C_{i}}{m_{\mathrm{AB}@Cu\text{-}MOF}}\times 100\%$$

where *E* represents the cumulative release rate; *V* denotes the volume of PBS replaced (1 mL); *V*_0_ is the total system volume (25 mL); *C*_n_ is the concentration of AB@Cu-MOF at the *n*-th sampling; *C*_i_ refers to the concentrations of AB@Cu-MOF from the first to the (*n* − 1)-th sampling; and *m*_AB@Cu-MOF_ is the loading amount of AB@Cu-MOF in hydrogel (mg).

### 2.17. Hemolysis Assay

Sheep blood was centrifuged at 1500 rpm for 5 min to collect sheep red blood cells (RBCs). After discarding the supernatant, the RBCs were repeatedly washed and centrifuged with PBS until the supernatant became colorless and finally resuspended in PBS to a constant volume of 1 mL. Equal volumes of PBS (negative control), H, AB@Cu-MOF, H + A@Cu-MOF, H + B@Cu-MOF, H + AB@Cu-MOF, and Triton X-100 (positive control) were added separately and incubated at 4 °C for approximately 4 h. After centrifugation, the supernatant was collected for absorbance detection at 540 nm via UV-vis spectrophotometer, and the hemolysis rate was calculated using Equation (5).

(5) $$\mathrm{Hemolysis}\left(\%\right)=\frac{A_{\mathrm{sample}\;}-{\;A}_{\mathrm{negative}}}{A_{\mathrm{positive}}\;-{\;A}_{\mathrm{negative}}}\times 100\%$$

### 2.18. Cell Culture

NIH3T3, PANC-1, and HPDE6-C7 cells were all purchased from the Cell Bank of the Chinese Academy of Sciences. All cells were routinely cultured in complete DMEM medium supplemented with 10% heat-inactivated FBS and 1% penicillin–streptomycin double antibiotics in an incubator containing 5% CO_2_ at 37 °C.

### 2.19. Biocompatibility Evaluation

The CCK-8 assay was adopted to evaluate the effects of H, AB@Cu-MOF and H + AB@Cu-MOF on the viability of NIH3T3 cells. NIH3T3 cells were seeded into 96-well plates at a density of approximately (4 × 10^3^) cells per well, and 100 μL of complete DMEM medium was added into each well for incubation at 37 °C with 5% CO_2_ for 24 h. Meanwhile, 1 mL of H and H + AB@Cu-MOF was separately immersed in 20 mL of complete DMEM medium for 24 h to prepare extraction solutions, which were further diluted with complete medium to obtain serial concentrations of 0%, 25%, 50%, 75%, and 100%. AB@Cu-MOF was directly dispersed in complete medium to prepare dispersions with concentrations of 0, 25, 50, 75, and 100 μg/mL. Cells were co-cultured with different extraction solutions or nano-dispersions for 24 h and 48 h, respectively. After removing the medium and rinsing with PBS, 100 μL of fresh medium and 10 μL of CCK-8 reagent were added to each well for further incubation for 1–4 h. The absorbance at 450 nm was measured using a microplate reader, and the relative cell viability was calculated accordingly.

### 2.20. Cytotoxicity Assay

CCK-8 assay and the Calcein-AM/PI double staining method were used to investigate the effects of various materials and treatment strategies on PANC-1 pancreatic cancer cells and HPDE6-C7 normal pancreatic epithelial cells. This experiment aimed to verify the targeted anti-tumor toxicity of the as-prepared materials and treatment regimens against pancreatic cancer cells as well as the synergistic therapeutic effect of nanocomposite hydrogels and assess their low cytotoxicity towards normal pancreatic cells to confirm biological safety. PANC-1 cells and HPDE6-C7 cells were evenly seeded into 24-well plates at a density of about 2 × 10^4^ cells per well, and 800 μL of complete DMEM medium was added for incubation at 37 °C in a 5% CO_2_ incubator for 24 h. Subsequently, Transwell inserts were placed above the 24-well plates, and 100 μL of corresponding hydrogel or nanoparticle solution was added into the inserts. After 6–8 h of co-culture with cells, different treatment interventions were implemented. The experimental groups were set as follows: (1) blank control group (Control); (2) hydrogel group (H); (3) H + AB@Cu-MOF group; (4) H + A@Cu-MOF group with 5 min of NIR irradiation (H + A@Cu-MOF + NIR); (5) H + B@Cu-MOF group with 5 min of NIR irradiation (H + B@Cu-MOF + NIR); (6) H + AB group with 5 min of NIR irradiation (H + AB + NIR); and (7) H + AB@Cu-MOF group with 5 min of NIR irradiation (H + AB@Cu-MOF + NIR). All NIR irradiation was performed at 808 nm with a power density of 1 W/cm^2^, and three replicate wells were arranged for each group. After the above treatments, cells were continuously cultured for another 24 h. Two replicate wells in each group were used for Calcein-AM/PI staining to observe cell survival status via fluorescence microscope. The supernatant collected from the remaining one replicate well after CCK-8 incubation was transferred into 96-well plates, and absorbance was detected using a microplate reader to calculate relative cell viability.

### 2.21. Intracellular Reactive Oxygen Species Detection

DCFH-DA was used as a fluorescent probe to detect intracellular reactive oxygen species (ROS) levels. PANC-1 cells were seeded into 24-well plates at 2 × 10^4^ cells per well and cultured with 800 μL of complete DMEM medium for 24 h. The experimental groups were listed as follows: (1) control; (2) H; (3) H + AB@Cu-MOF; (4) H + A@Cu-MOF + NIR; (5) H + AB + NIR; (6) H + AB@Cu-MOF + NIR; and (7) ROSup. All NIR irradiation was conducted at 808 nm and 1 W/cm^2^. As a positive reagent for inducing ROS generation, ROSup could trigger massive intracellular ROS production within 20–30 min of administration. Except for when ROSup was added 30 min before observation, hydrogels in other groups were co-cultured with cells for 6 h firstly, followed by corresponding NIR treatment and further incubation for 10 h. Afterwards, the culture medium was discarded, and cells were rinsed with PBS. Then, 10 μL of DCFH-DA working solution was added for light-proof incubation for 30 min. After removing unbound probes through PBS washing, intracellular ROS generation was observed and recorded under a fluorescence microscope.

### 2.22. Establishment of Subcutaneous Tumor-Bearing Mouse Model

Female Balb/c-nu nude mice aged 3–5 weeks were purchased from Shanghai SLAC Laboratory Animal Co., Ltd., (Shanghai, China). All animal experiments were approved by the Ethics Committee of Tongji University (Ethics Approval No.: TJIA2026035) and strictly carried out in accordance with the National Regulations for the Administration of Laboratory Animals of China. A total of 50 μL of PANC-1 cell suspension containing approximately 1 × 10^7^ cells was subcutaneously injected into the right axilla of each nude mouse. After 7 days of feeding, the tumor volume reached about 100 mm^3^, indicating successful establishment of tumor-bearing models. The tumor volume V was calculated according to Equation (6).

V = L × W^2^ × 0.5 (6)

where L represents the maximum tumor diameter and W represents the minimum diameter perpendicular to L.

### 2.23. In Vivo Tumor Inhibition

Successfully modeled nude mice were randomly divided into six groups with five mice in each group. The sample size for animal experiments was determined based on statistical power analysis, which verifies that five mice per group is sufficient to detect intergroup differences. Detailed calculation procedures and formulas are provided in Text S1. A total volume of 50 μL of different hydrogels or nanoparticle materials was directly injected into tumor sites, followed by corresponding treatments. The specific grouping was as follows: (1) PBS group; (2) H + AB@Cu-MOF group; (3) H + A@Cu-MOF + NIR group; (4) H + B@Cu-MOF + NIR group; (5) H + AB + NIR group; and (6) H + AB@Cu-MOF + NIR group. All NIR irradiation parameters were set as 808 nm and 1 W/cm^2^. NIR irradiation was performed for 5 min each time on Groups (3), (4), (5), and (6) on Days 1, 3, 5, 7, and 9 after treatment. An infrared thermal imager was used to record temperature changes in tumor areas. Meanwhile, the body weights of mice and tumor volume were measured regularly. All mice were sacrificed on Day 13 after treatment. Tumor tissues and major visceral organs, including heart, liver, spleen, lung, and kidney, were isolated for photographing and weighing. All tissue samples were fixed in 4% paraformaldehyde and stored at 4 °C for subsequent paraffin embedding, sectioning, H&E staining, and TUNEL staining analysis.

### 2.24. Statistical Analysis

All data obtained from cell and animal experiments were analyzed through one-way analysis of variance (ANOVA) followed by Tukey’s post hoc test for multiple comparisons. All results are presented as mean ± standard deviation (mean ± SD). The statistical significance was defined as * *p* < 0.05, ** *p* < 0.01, and *** *p* < 0.001.

## 3. Results

### 3.1. Preparation of AB@Cu-MOF

**Figure 1:**
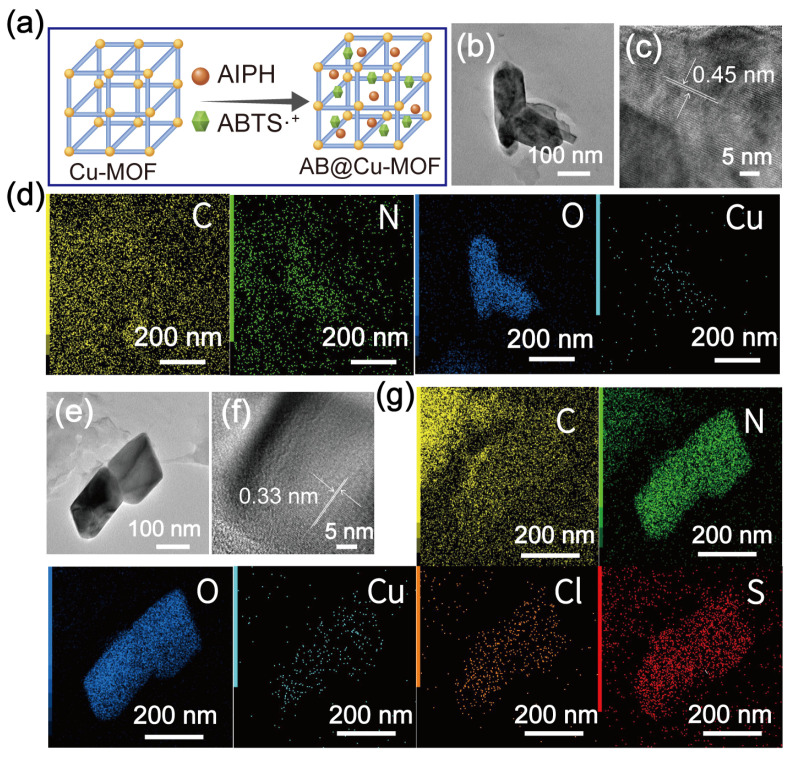
(**a**) Structural schematic diagrams of Cu-MOF and AB@Cu-MOF. (**b**) TEM image of Cu-MOF. (**c**) High-resolution TEM image of Cu-MOF. (**d**) Elemental mapping images of Cu-MOF (C, N, O, Cu). (**e**) TEM image of AB@Cu-MOF. (**f**) High-resolution TEM image of AB@Cu-MOF. (**g**) Elemental mapping images of AB@Cu-MOF (C, N, O, Cu, Cl, S).

AB@Cu-MOF was synthesized via a solvothermal method. After stirring treatment, AIPH and ABTS·^+^ were loaded onto the framework of Cu-MOF. Figure 1a illustrates the above synthetic process. Figure 1b displays the TEM image of pure Cu-MOF, which presents regular prismatic nanocrystal morphology with uniform particle sizes ranging from 200 to 300 nm. The high-resolution TEM image in Figure 1c clearly shows continuous and ordered lattice fringes with an interplanar spacing of approximately 0.45 nm, verifying its favorable crystallinity and intact crystal structure. The elemental mapping results in Figure 1d reveal the homogeneous distribution of C, N, O, and Cu elements within Cu-MOF, which are well matched with the nanocrystal morphology. Figure 1e shows the TEM image of AB@Cu-MOF after loading AIPH and ABTS·^+^. It still retains the identical prismatic nanocrystal shape of pristine Cu-MOF, with particle sizes staying within 200–300 nm. The high-resolution TEM image in Figure 1f exhibits distinct and intact lattice fringes of AB@Cu-MOF with an interplanar spacing of about d = 0.33 nm, which is slightly smaller than that of pure Cu-MOF. This phenomenon may originate from the loaded small molecules, slight adjustment of the coordination environment of MOF, or minor lattice strain, while it exerts no obvious influence on the crystallinity of Cu-MOF. The elemental mapping results in Figure 1g confirm that apart from the inherent C, N, O, and Cu elements of Cu-MOF, Cl (characteristic element of AIPH) and S (characteristic element of ABTS·^+^) are uniformly distributed over the nanocrystal region and highly consistent with the main morphology. This directly demonstrates that AIPH and ABTS·^+^ are successfully and evenly immobilized on the Cu-MOF skeleton and that the AB@Cu-MOF nanocomposite is well constructed. Quantitative results revealed that the encapsulation efficiency of AIPH and ABTS·^+^ was 64.53% and 70.35% and their loading efficiency was 38.72% and 42.21%, respectively. As a porous carrier, Cu-MOF exhibited excellent loading capacity for both active molecules. Sufficient loading of functional components guaranteed their adequate concentration in the therapeutic system, which laid an important foundation for the material to achieve high-efficiency triple synergistic anti-tumor effects of PTT, TDT, and CDT.

**Figure 2:**
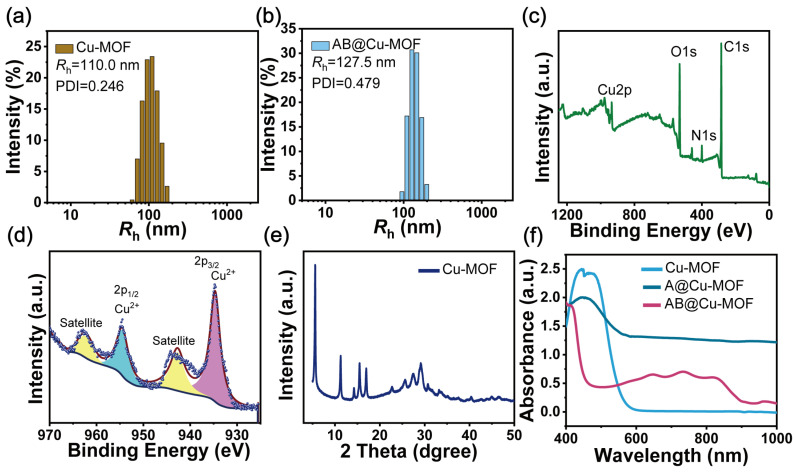
(**a**) Hydrodynamic radius (*R*_h_) of Cu-MOF. (**b**) *R*_h_ of AB@Cu-MOF. (**c**) Full-range XPS spectrum of Cu-MOF. (**d**) High-resolution XPS spectrum of Cu 2p in Cu-MOF. (**e**) XRD pattern of Cu-MOF. (**f**) UV-vis absorption spectra of Cu-MOF, A@Cu-MOF, and AB@Cu-MOF.

DLS measurements show that the average hydrodynamic radii of Cu-MOF and AB@Cu-MOF are 110.0 nm (PDI = 0.246) and 127.5 nm (PDI = 0.479), respectively (Figure 2a,b), which are basically consistent with the particle sizes obtained from TEM results. The loading of AIPH and ABTS·^+^ leads to a slight increase in particle size. Figure 2c presents the full-range XPS spectrum of Cu-MOF, in which characteristic peaks of C1s, N1s, O1s, and Cu2p are distinctly detected, confirming that the elemental composition of as-synthesized Cu-MOF is in good accordance with the theoretical design. The high-resolution Cu2p spectrum is shown in Figure 2d. After peak fitting, prominent characteristic peaks assigned to Cu^2+^, including 2p3/2 (~934.6 eV), 2p1/2 (~954.5 eV), and obvious satellite peaks, can be observed. The abundant Cu^2+^ can efficiently consume high-concentration GSH in the tumor microenvironment via redox reactions, thus preventing the premature reduction and deactivation of ABTS·^+^ by GSH and ensuring a sustainable photothermal effect in subsequent applications. The survey XPS spectrum of AB@Cu-MOF (Figure S1) displays S 2p (~169 eV) and Cl 2p (~198 eV) signals, verifying the successful loading of ABTS·^+^ and AIPH. The XRD pattern of Cu-MOF is shown in Figure 2e. The sharp characteristic diffraction peaks indicate its excellent crystallinity, which is highly consistent with the standard pattern of target Cu-MOF, further proving the successful synthesis and high purity of Cu-MOF [63]. Figure 2f shows the UV-Vis absorption spectra of Cu-MOF, A@Cu-MOF, and AB@Cu-MOF. Pure Cu-MOF exhibits characteristic absorption bands in the range of 400–550 nm, and A@Cu-MOF possesses almost identical spectral features. In contrast, AB@Cu-MOF displays a broad characteristic absorption peak of ABTS·^+^ within 600–900 nm, which directly verifies the successful loading of ABTS·^+^.

### 3.2. Preparation of APMOH/CMCS Hydrogel

**Figure 3:**
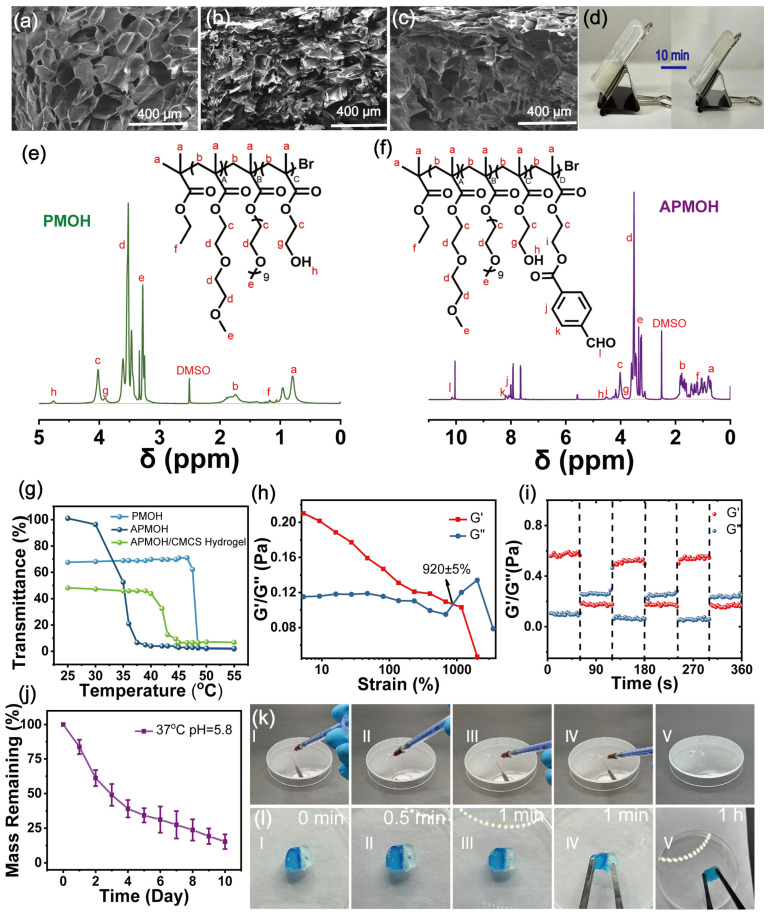
(**a**) SEM image of APMOH/CMCS hydrogel. (**b**) SEM image of APMOH/CMCS/AB@Cu-MOF nanocomposite hydrogel. (**c**) SEM image of hydrogel after heating above T_VPT_ and rapid fixation via liquid nitrogen quenching. (**d**) Gelation process of APMOH/CMCS hydrogel. ^1^H NMR spectra of (**e**) PMOH and (**f**) APMOH. (**g**) Temperature–transmittance curves of PMOH, APMOH and APMOH/CMCS hydrogel. (**h**) Shear strain sweep curve of APMOH/CMCS hydrogel. (**i**) Continuous step–strain test curve of APMOH/CMCS hydrogel. (**j**) Degradation curve of APMOH/CMCS hydrogel at 37 °C and pH 5.8. (**k**) Photograph of APMOH/CMCS hydrogel injected through a 26G needle (Images (**I**–**V**) demonstrate the process of writing the two letters “jh” using a syringe needle). (**l**) Photographs verifying the self-healing performance of APMOH/CMCS hydrogel (Images (**I**–**III**) display the states of the self-healing hydrogel at 0, 0.5, and 1 min, respectively. Images (**IV**,**V**) show the tensile states of the hydrogel pulled with tweezers after self-healing for 1 min and 1 h).

APMOH serves as the core matrix material for constructing the three-dimensional network of hydrogels. The MEO_2_MA and OEGMA units on its molecular chains endow the system with prominent thermoresponsive properties. Meanwhile, aldehyde functionalization modification is performed on the side chains of HEMA. The aldehyde groups on APMOH can undergo a condensation reaction with amino groups on CMCS molecules, thereby forming hydrogel networks crosslinked via imine bonds. As typical dynamic covalent bonds, imine bonds can achieve reversible cleavage and reconstruction under external stimuli [65]. This unique chemical property endows APMOH/CMCS hydrogels with excellent injectability, self-healing capacity, and thermosensitive behavior. Relevant tests were carried out to characterize the structure and properties of APMOH/CMCS hydrogels. Figure 3a shows the SEM image of pristine APMOH/CMCS hydrogel, which possesses uniformly interconnected three-dimensional porous network structures. Figure 3b displays APMOH/CMCS hydrogel loaded with AB@Cu-MOF, whose inherent three-dimensional porous structure is well-preserved, confirming that the loading of small molecules does not damage the intrinsic morphology of hydrogels. APMOH/CMCS hydrogels exhibit typical thermoresponsive characteristics. Figure 3c presents the SEM image of hydrogels heated above T_VPT_ and rapidly frozen with liquid nitrogen for structural fixation. It can be observed that the three-dimensional network structure obviously collapses, which directly verifies the thermoresponsive behavior of such thermosensitive hydrogels and provides a structural basis for their application as stimuli-responsive carriers to realize controlled release of loaded substances. Figure 3d shows the digital photograph of APMOH/CMCS hydrogel. After uniform mixing of APMOH and CMCS solutions for 10 min, the mixture transforms from sol state to gel state without flowing when inverted. Based on integral calculations from the ^1^H NMR spectra of PMOH and APMOH shown in Figure 3e,f, the molar ratio of MEO_2_MA segments to OEGMA segments in PMOH is approximately 3.9:1, and the molar ratio of total MEO_2_MA and OEGMA segments to HEMA segments is about 16.0:1. The barely observable signal of hydroxyl protons in the APMOH spectrum indicates a high degree of aldehyde substitution, which can be attributed to the sufficient feeding amount of the aldehyde precursor and the nearly complete substitution reaction. The detailed calculation of the aldehyde substitution degree is described as follows: Peak *d* from the polymer backbone (inactive during esterification modification) was chosen as the universal internal reference. For the spectrum displayed in Figure 3e, the integral ratio of peak *h* (hydroxyl protons) to internal reference peak *d* was calculated to be 0.0166. For the spectrum displayed in Figure 3f, the integral ratio of peak *l* (aldehyde protons) to internal reference peak *d* was determined to be 0.0164. Accordingly, the aldehyde substitution degree is calculated as (0.0164/0.0166) × 100% ≈ 98.8%. FT-IR spectra of PMOH, APMOH, and APMOH/CMCS in Figure S2 further verify the successful aldehyde functionalization and effective crosslinking of hydrogels. GPC data for PMOH and APMOH are shown in Figure S3. PMOH exhibits a single narrow unimodal peak free of low-molecular oligomers with number-average molecular weight *M*_n_ = 18,170 g/mol, confirming well-controlled ATRP and uniform polymer chains. After aldehyde grafting modification, the *M*_n_ of APMOH increased to 25,620 g/mol, alongside a slight PDI reduction from 1.59 to 1.57 (PDI = weight-average molecular weight *M*_w_/*M*_n_). No intermediate oligomeric peaks appear between the polymer and small-molecule signals, ruling out polymer heterogeneity and backbone degradation. Figure 3g depicts the temperature–transmittance curves of PMOH, APMOH, and APMOH/CMCS hydrogels, which clearly reflect their thermoresponsive behaviors. The transmittance of PMOH remains stable below approximately 48 °C and drops sharply as the temperature approaches 48 °C, showing typical thermosensitive phase transition behavior. This phenomenon originates from the thermosensitivity of MEO_2_MA and OEGMA units. Nevertheless, weak hydrophobic interactions exist between the alkyl backbones of polymer chains even under fully dissolved conditions, leading to the generation of trace transient nano-sized hydrophobic clusters at room temperature that cause mild light scattering, so the baseline transmittance of PMOH only stabilizes at around 70%. Once the temperature exceeds the T_VPT_, intermolecular hydrogen bonds dissociate, and polymer chains undergo massive hydrophobic aggregation, resulting in drastic turbidity and a sharp decline in transmittance. After aldehyde modification, the T_VPT_ of APMOH is significantly reduced to about 35 °C. The introduction of aldehyde groups increases the hydrophobicity of polymers and weakens hydrogen bonding interactions between molecular chains and water molecules, enabling hydrophobic aggregation to occur at lower temperatures. The T_VPT_ of APMOH/CMCS hydrogels is further adjusted to around 43 °C, between those of PMOH and APMOH. The hydrophilic groups of CMCS enhance the hydrophilicity of the system, leading to a slight increase in T_VPT_ compared with pure APMOH. In addition, the steric hindrance of crosslinked networks restricts the free movement of molecular chains, resulting in a slightly wider phase transition interval than that of pure polymers. The variation trend of T_VPT_ among the three samples is consistent with chemical structural modification and crosslinking effects, proving that the thermoresponsive performance of hydrogels can be precisely regulated by adjusting composition and structure, which lays a theoretical foundation for their application as temperature-responsive drug delivery carriers. Figure S4 shows digital photographs of H + AB@Cu-MOF hydrogels before and after NIR irradiation. Under NIR irradiation, photothermal agent ABTS·^+^ inside of hydrogels induces photothermal effects to raise the system’s temperature and trigger volume shrinkage of hydrogels. Meanwhile, the hydrophilic–hydrophobic transition of polymer chains causes exudation of internal liquid from hydrogels. Figure 3h,i illustrate the rheological properties of APMOH/CMCS hydrogels to confirm their shear-thinning behavior and self-healing performance. Strain sweep results (Figure 3h) reveal that the storage modulus G′ is much higher than the loss modulus G″ within the low strain range, indicating solid-like elastic behavior of hydrogels. When the strain increases to approximately 920%, G′ drops sharply and becomes lower than G″, suggesting the transition of hydrogels from elastic solids to viscous fluids. This critical strain value indicates that the hydrogel possesses moderate viscosity and injection resistance under shear force during administration, enabling convenient clinical puncture and injection. It can effectively avoid material leakage caused by excessively low viscosity and needle blockage resulting from overly high viscosity. In addition, the maximum extrusion pressure of the hydrogel during slow injection through a 26G needle was approximately 102.4 ± 4.7 kPa, which further confirms its moderate injection pressure. Alternating strain sweep tests further confirm the self-healing capability of hydrogels (Figure 3i). After being damaged under 1000% large strain, G′ decreases drastically. When switched back to 1% low strain, G′ recovers rapidly and approaches the initial value, demonstrating that dynamic covalent bonds and reversible interactions can realize rapid reconstruction of network structures and endow materials with outstanding self-repairing performance. Figure 3j shows the in vitro degradation curve of APMOH/CMCS hydrogels at 37 °C and pH 5.8. The results indicate that the residual mass of hydrogels gradually decreases with prolonged degradation time, with a residual mass ratio of about 15.3% on the 10th day, demonstrating a favorable degradation rate. Such degradation behavior is attributed to the hydrolytic cleavage of Schiff base bonds in hydrogels as well as the swelling and degradation of CMCS itself in acidic environments, which matches the physiological microenvironment in vivo and the treatment cycle of tumor therapy. The injectability of APMOH/CMCS hydrogels is verified via injection using a 26G needle. The hydrogels can be extruded smoothly and continuously without blockage or fracture (Figure 3k). Figure 3l characterizes the self-healing performance of hydrogels. Cylindrical hydrogels with a diameter of about 1 cm and a height of about 0.2 cm are cut into two halves, one of which is dyed and then closely fitted together. The color boundary gradually blurs after standing for 0.5 min and 1 min, indicating the initiation of interfacial self-healing. The healed hydrogel can withstand gentle traction with tweezers without separation after 1 min, proving its rapid and superior self-healing ability. After standing for 1 h, the blue dye fully diffuses throughout the reconstructed hydrogel, which still maintains structural integrity under traction, further confirming the stable self-healing network structure. The excellent self-healing capability enables the hydrogel to rapidly reconstruct its three-dimensional network structure after injection and mechanical disturbance within tumor tissues. This effectively prevents material leakage at the injection site, maintains the retention of therapeutic components in tumor regions, and thereby reduces off-target toxic and side-effects. From the perspective of clinical translation, however, this system still faces practical challenges. Given the deep location of pancreatic tumors, professional imaging equipment is required to guide injection and achieve accurate positioning.

### 3.3. Self-Regeneration Performance of AB@Cu-MOF

**Figure 4:**
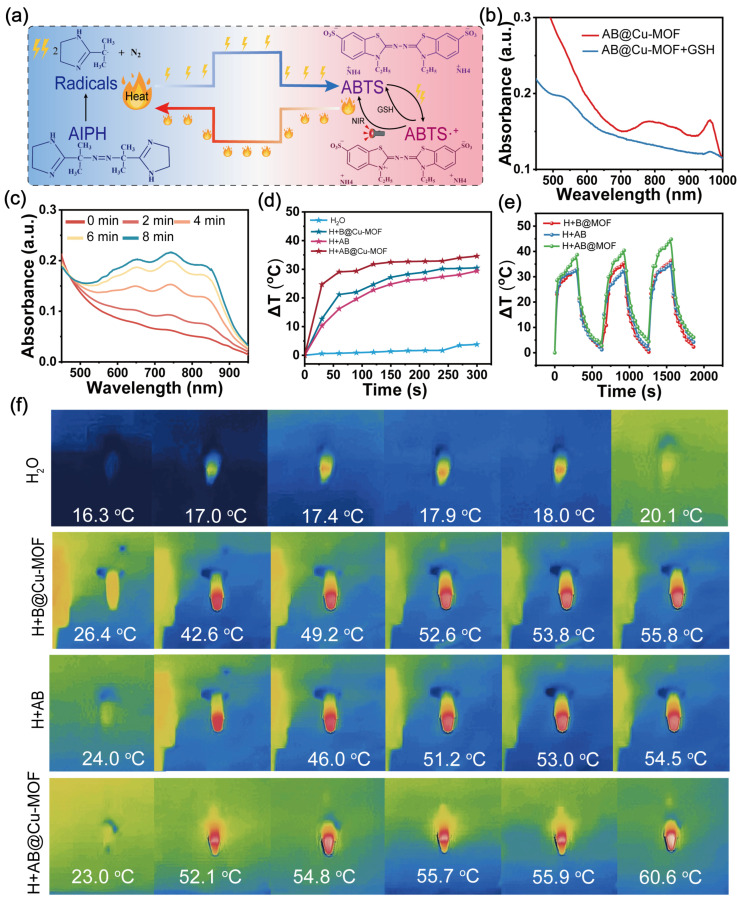
(**a**) Schematic diagram of PTT-TDT synergistic therapy and ABTS·^+^ self-regeneration mechanism of AB@Cu-MOF. (**b**) UV-vis absorption spectra of AB@Cu-MOF before and after GSH addition. (**c**) Time-dependent UV-vis absorption spectra of GSH-pretreated AB@Cu-MOF under 808 nm of NIR irradiation at 1 W/cm^2^. (**d**) Temperature elevation curves of H_2_O, H + B@Cu-MOF, H + AB, and H + AB@Cu-MOF within 5 min under 808 nm of NIR irradiation at 1 W/cm^2^. (**e**) Three cycles of heating and cooling of H + B@Cu-MOF, H + AB, and H + AB@Cu-MOF under 808 nm of NIR irradiation at 1 W/cm^2^. (**f**) Infrared thermal imaging images showing photothermal temperature rise of H_2_O, H + B@Cu-MOF, H + AB, and H + AB@Cu-MOF.

Figure 4a illustrates the PTT-TDT synergistic therapy and ABTS·^+^ self-regeneration mechanism of AB@Cu-MOF. Under NIR irradiation, ABTS·^+^ acts as a photothermal agent to achieve efficient photothermal conversion and elevate ambient temperature for PTT. Meanwhile, the temperature increase triggers the decomposition of AIPH to release alkyl radicals for TDT. High-concentration GSH in the tumor microenvironment can reduce ABTS·^+^ and weaken the photothermal effect. In contrast, alkyl radicals generated from AIPH can reoxidize reduced ABTS back into ABTS·^+^, thus realizing the self-regeneration of photothermal agents and ensuring sustainable PTT-TDT synergistic treatment. Figure 4b confirms the consumption effect of GSH on ABTS·^+^. After adding GSH, the characteristic absorption peak of ABTS·^+^ at 734 nm of AB@Cu-MOF obviously decreases, proving that GSH can reduce ABTS·^+^. The UV-Vis spectra in Figure 4c directly verify the self-regeneration capacity of ABTS·^+^. Upon 808 nm NIR irradiation at 1 W/cm^2^, the absorbance at 734 nm of GSH-treated AB@Cu-MOF rises continuously within 0–8 min. Addition of L-ascorbic acid scavenges alkyl radicals in the system and thus inhibits the regeneration of ABTS·^+^, and this inhibitory effect is concentration-dependent. Compared with the group containing 0.2 mg/mL of L-ascorbic acid and the control group without radical scavengers, the group with 0.8 mg/mL of L-ascorbic acid exhibited a distinctly slower increase in absorbance at 734 nm, indicating that a higher concentration of scavengers corresponds to a lower generation rate of ABTS·^+^. These results confirm that alkyl radicals generated from the decomposition of AIPH are the key active species responsible for the regeneration of ABTS·^+^ (Figure S5).

### 3.4. Photothermal Performance Measurement and Thermal Imaging of Nanocomposite Hydrogels

As shown in Figure 4d, under 808 nm of NIR irradiation at 1 W/cm^2^, both H + B@Cu-MOF and H + AB exhibited rapid temperature elevation within 5 min, with final temperature increases (ΔT) of 30.5 °C and 29.4 °C, respectively. By comparison, H + AB@Cu-MOF achieved a faster heating rate and higher final ΔT of 34.6 °C, which was distinctly superior to the other two groups. This confirms the synergistic effect between AIPH and ABTS·^+^. Alkyl radicals produced through the thermal decomposition of AIPH can oxidize reduced ABTS to replenish inactivated ABTS·^+^ and sustain high photothermal efficiency. Meanwhile, Cu-MOF alleviates GSH-induced consumption of ABTS·^+^ to further enhance photothermal performance. No obvious temperature rise was observed in the H_2_O group, ruling out the intrinsic photothermal effect of the hydrogel matrix. Infrared thermal images in Figure 4f visually present the photothermal heating differences among H_2_O, H + B@Cu-MOF, H + AB, and H + AB@Cu-MOF, which are consistent with the temperature rise curves. Cyclic stability tests in Figure 4e reveal that the ΔT values of all three samples remained stable after three successive rounds of 808 nm NIR irradiation at 1 W/cm^2^ (H + B@Cu-MOF: 32.6 °C, 35.4 °C, 36.4 °C; H + AB: 33.0 °C, 32.6 °C, 35.2 °C; H + AB@Cu-MOF: 38.7 °C, 40.4 °C, 44.8 °C), demonstrating satisfactory photothermal stability suitable for repeated in vivo photothermal therapy. The slight temperature rise during cycles is attributed to local enrichment of photothermal agents caused by water evaporation from heated hydrogels. The consistently higher ΔT of H + AB@Cu-MOF throughout cycles further verifies that AIPH and Cu-MOF jointly boost the photothermal effect of ABTS·^+^. After multiple cycles of NIR irradiation, the local temperature elevation presents favorable repeatability. The peak temperature in the irradiated area remains stable with no abnormal heat accumulation, thus ensuring thermal safety during repeated treatments. Figure S6 displays three photothermal cycling curves and fitted time constant (τ) results of H + B@Cu-MOF, H + AB, and H + AB@Cu-MOF. A larger τ value indicates slower heat dissipation and better heat storage capacity. The τ values of H + AB@Cu-MOF were remarkably higher than those of the other two groups and stayed stable in repeated cycles, suggesting its outstanding heat storage capability and photothermal durability. This guarantees efficient and long-lasting photothermal therapy as well as thermo-responsive shrinkage and controlled release of hydrogel carriers.

### 3.5. Photothermally Enhanced Thermodynamic Performance

**Figure 5:**
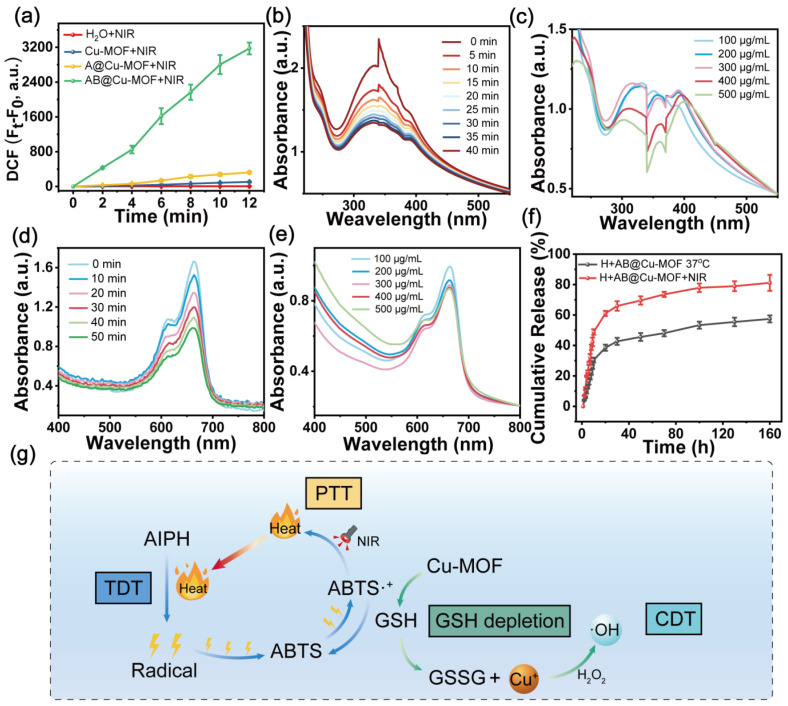
(**a**) DCF fluorescence intensity variation curves of H_2_O, Cu-MOF, A@Cu-MOF, and AB@Cu-MOF under 808 nm of NIR irradiation at 1 W/cm^2^ for 12 min. (**b**) Time-dependent UV-vis absorption spectra of DTNB degradation at 412 nm in the presence of 500 μg/mL of Cu-MOF. (**c**) Variation curve of DTNB degradation degree versus Cu-MOF concentration at identical reaction time. (**d**) UV-vis absorption spectra of MB at 664 nm after reaction between GSH-pretreated AB@Cu-MOF and H_2_O_2_ using MB as ·OH trapping probe. (**e**) Absorbance variation curves of MB at 664 nm with different Cu-MOF concentrations after the same reaction duration. (**f**) Cumulative release curves of H + AB@Cu-MOF hydrogel under different conditions. (**g**) Schematic diagram of synergistic therapeutic mechanism of AB@Cu-MOF.

The continuous regeneration of ABTS·^+^ in AB@Cu-MOF is attributed to the efficient oxidation of ABTS by alkyl radicals derived from the thermal decomposition of AIPH. DCFH-DA was used as a fluorescent probe for real-time monitoring of the production of alkyl radicals. As shown in Figure 5a, the DCF fluorescence intensity increased remarkably when GSH-pretreated AB@Cu-MOF was irradiated with 808 nm of NIR light at 1 W/cm^2^ for 12 min, indicating that photothermal-induced temperature elevation greatly accelerated AIPH decomposition and triggered massive alkyl radical generation. In control groups, neither H_2_O nor pure Cu-MOF showed obvious fluorescence response under the same conditions, confirming that the substrate materials cannot produce radicals. Only a slight fluorescence enhancement was observed for A@Cu-MOF, which was caused by trace radicals generated via the NIR thermal effect.

### 3.6. GPx-like Catalytic Activity of Cu-MOF

DTNB was adopted as a probe to capture GSH and verify the GSH scavenging capacity of Cu-MOF. As illustrated in Figure 5b, the absorbance of the characteristic absorption peak at 412 nm gradually decreased with the reaction time ranging from 0 to 40 min, which indicated the gradual consumption of GSH and proved the prominent GPx-like catalytic activity of Cu-MOF. As the carrier for loading AIPH and ABTS·^+^, Cu-MOF can pre-consume excessive GSH in the tumor microenvironment to prevent premature reduction and deactivation of ABTS·^+^, thereby ensuring the effective initiation of combined PTT-TDT therapy. Figure 5c presents the GSH consumption results of Cu-MOF at different concentrations. Within the concentration range of 100–500 μg/mL, the GSH consumption rate increased significantly with the rise in Cu-MOF concentration, demonstrating its efficient and tunable GSH scavenging ability to effectively maintain the photothermal activity of ABTS·^+^.

### 3.7. POD-like Catalytic Activity of AB@Cu-MOF

Cu-MOF can be reduced to Cu^+^ after consuming GSH. Methylene blue (MB) was used as the ·OH trapping probe to verify the Fenton-like performance of Cu^+^ for catalyzing H_2_O_2_ to produce hydroxyl radicals. As shown in Figure 5d, the absorbance of the MB characteristic peak at 664 nm declined continuously over the 0–50 min reaction period, which confirmed that GSH-pretreated AB@Cu-MOF could generate abundant Cu^+^ and further produce hydroxyl radicals efficiently through Fenton-like reactions. Figure 5e shows the ·OH generation results of AB@Cu-MOF with varied concentrations. In the range of 100–500 μg/mL, the ·OH production rate obviously increased with increasing material concentration, revealing the concentration-dependent POD-like catalytic activity of AB@Cu-MOF for efficient CDT. The synergistic therapeutic mechanism of AB@Cu-MOF is displayed in Figure 5g.

### 3.8. Controlled Release Behavior of Nanocomposite Hydrogels

Figure 5f shows the cumulative release profiles of encapsulated substances from H + AB@Cu-MOF hydrogels under different conditions. Benefiting from the thermoresponsive phase transition property of the APMOH/CMCS hydrogel matrix, the hydrogel maintains its intact network structure without phase separation or structural collapse at the physiological temperature of 37 °C, leading to a slow release rate and a low cumulative release ratio of approximately 57.4%, which endows the system with a favorable passive targeting effect and long-term retention capability. Under NIR irradiation, local temperature was sharply elevated above 43 °C via the photothermal effect, which further triggered rapid volume phase transition and structural collapse of APMOH/CMCS hydrogels. The destroyed dense network structure greatly accelerated drug release, and the final cumulative release rate reached up to 81.1%. The above results confirm that H + AB@Cu-MOF nanocomposite hydrogels possess excellent NIR-triggered controlled release performance, which can realize on-demand precise drug release responding to thermal stimuli in the tumor microenvironment and effectively reduce toxic side-effects on normal tissues.

### 3.9. Biocompatibility of Nanocomposite Hydrogels

**Figure 6:**
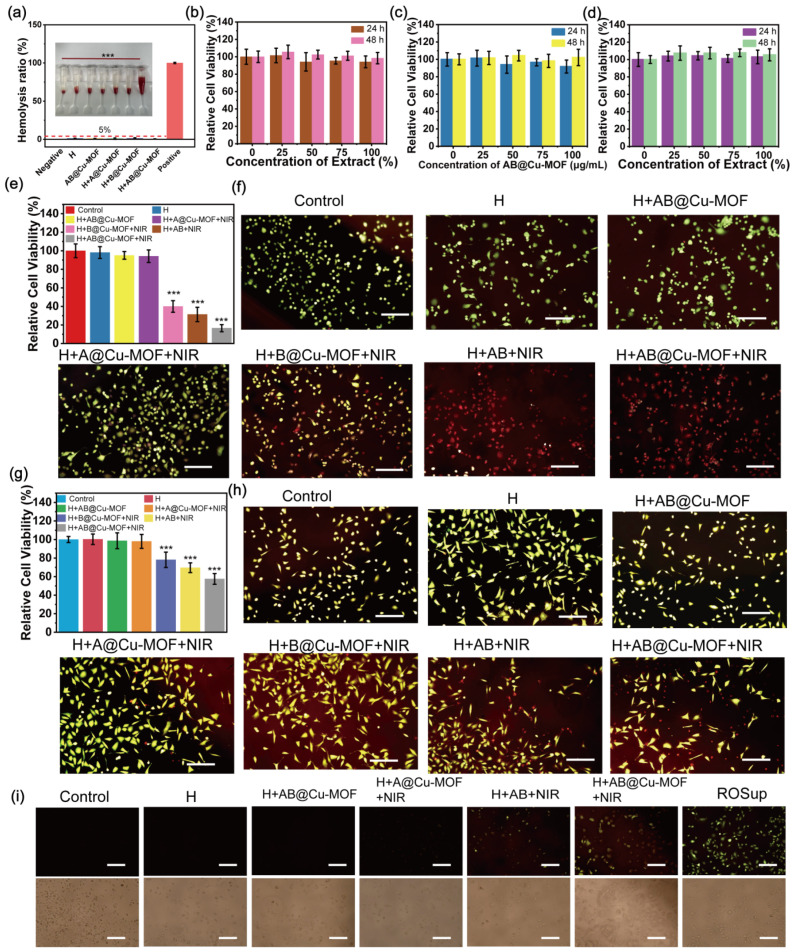
(**a**) Hemolysis photographs and hemolysis rates of each group (Negative, H, AB@Cu-MOF, H + A@Cu-MOF, H + B@Cu-MOF, H + AB@Cu-MOF, Positive) (*n* = 3, mean ± SD, *** *p* < 0.001). (**b**) Relative viabilities of NIH3T3 cells after incubation with H group, (**c**) AB@Cu-MOF group, and (**d**) H + AB@Cu-MOF group for 24 h and 48 h, respectively (*n* = 8, mean ± SD). (**e**) Relative viabilities of PANC-1 cells under different treatments (*n* = 8, mean ± SD, *** *p* < 0.001). (**f**) Fluorescence microscopic images of live/dead PANC-1 cells stained with Calcein-AM/PI kit under different treatments (scale bar: 200 μm). (**g**) Relative viabilities of HPDE6-C7 cells under different treatments (*n* = 8, mean ± SD, *** *p* < 0.001). (**h**) Fluorescence microscopic images of live/dead HPDE6-C7 cells stained with Calcein-AM/PI kit under different treatments (scale bar: 200 μm). (**i**) Fluorescence microscopic images showing DCFH-DA-labeled ROS generation under different treatments (scale bar: 200 μm).

To evaluate the in vivo application potential of H + AB@Cu-MOF nanocomposites, a hemolysis assay and a CCK-8 cell viability assay were conducted to systematically verify their biocompatibility. As shown in Figure 6a, the hemolysis rates of all experimental groups, including H, AB@Cu-MOF, H + A@Cu-MOF, H + B@Cu-MOF, and H + AB@Cu-MOF, were far below the safety threshold of 5%, showing no significant difference from the negative control group. Obvious hemolysis was observed in the positive control group treated with Triton X-100, which confirmed that all prepared materials possessed excellent blood compatibility without causing erythrocyte rupture and damage. CCK-8 assay was further adopted to assess cytotoxicity against normal NIH3T3 cells. The results revealed that after 24 h and 48 h of co-culture, cell viability remained above 90% when incubated with hydrogel extracts at concentrations ranging from 0% to 100% and AB@Cu-MOF dispersions at concentrations from 0 to 100 μg/mL, with no obvious concentration-dependent decline (Figure 6b,c). Similarly, extracts of H + AB@Cu-MOF exhibited favorable cytocompatibility at all tested concentrations without remarkable reduction in cell viability within 48 h (Figure 6d). These results fully demonstrate that both H hydrogel matrix and AB@Cu-MOF nanomaterials have superior biological safety, which lays a solid foundation for their further application in in vivo anti-tumor therapy.

### 3.10. In Vitro Anti-Tumor Activity and Cell Selectivity of Nanocomposite Hydrogels

CCK-8 assay and Calcein-AM/PI double staining were applied to explore the synergistic anti-tumor effect and cell selectivity of H + AB@Cu-MOF hydrogels by evaluating the cytotoxicity of different treatment strategies toward PANC-1 pancreatic cancer cells and HPDE6-C7 normal pancreatic epithelial cells. According to the CCK-8 cytotoxicity data in Figure 6e, the relative viabilities of PANC-1 cells in the blank control group and the pure H hydrogel group were both higher than 98%, proving that the hydrogel matrix had negligible cytotoxicity and good biocompatibility. The non-irradiated H + AB@Cu-MOF group only induced mild cell damage with cell viability of approximately 95%. This phenomenon was attributed to the limited intracellular GSH content in in vitro culture systems, which weakened the Cu-MOF-mediated Fenton-like CDT effect and failed to achieve effective cell killing. Under NIR irradiation, the H + A@Cu-MOF + NIR group only containing AIPH showed a weak therapeutic effect with cell viability of about 94% due to insufficient alkyl radical production and limited photothermal performance. The H + B@Cu-MOF + NIR group only containing ABTS·^+^ possessed moderate PTT capability yet lacked continuous alkyl radical supply for synergistic treatment, resulting in a restricted anti-tumor effect and a cell viability of around 40%. The cell viability of the H + AB + NIR group was approximately 31%. In the absence of Cu-MOF carriers, efficient GSH depletion and Fenton-like reactions could not be activated, leading to the loss of the CDT effect. Meanwhile, premature inactivation of ABTS·^+^ further impaired the cyclic therapeutic effect of combined PTT and TDT. In contrast, the H + AB@Cu-MOF + NIR group realized triple synergistic therapy, including PTT based on ABTS·^+^ photothermal conversion, TDT relying on AIPH-derived alkyl radicals, and CDT via Cu-MOF-catalyzed hydroxyl radical generation. This combined treatment markedly strengthened anti-tumor capacity and decreased relative cell viability to 16%, presenting optimal anti-cancer efficacy. The Calcein-AM/PI double staining results in Figure 6f were highly consistent with the CCK-8 results. In the H + AB@Cu-MOF + NIR group, green fluorescence representing living cells almost disappeared, while red fluorescence of dead cells dominated the visual field, directly verifying the potent anti-tumor ability of this synergistic therapeutic strategy. Cytotoxicity results against normal HPDE6-C7 cells are displayed in Figure 6g. Even for the H + AB@Cu-MOF + NIR group with the strongest killing effect, nearly 57% cell viability was maintained, and the proportion of living cells was distinctly higher than that of PANC-1 cells, confirmed through Calcein-AM/PI staining. The intracellular GSH level in normal cells is much lower than that in tumor microenvironments, which greatly inhibits GSH scavenging behavior and Fenton-like reactions of Cu-MOF. In addition, the activation of ABTS·^+^ and AIPH is dependent on high GSH concentrations and photothermal stimulation within tumor tissues. Such characteristics enable targeted elimination of tumor cells and minimize side-effects on normal tissues. The Calcein-AM/PI staining results in Figure 6h were consistent with CCK-8 data. All of the above findings confirm that H + AB@Cu-MOF hydrogels can effectively eliminate pancreatic cancer cells via triple PTT/TDT/CDT synergistic effects with outstanding cell selectivity and biological safety.

### 3.11. Intracellular Radical Detection

A DCFH-DA fluorescent probe was used to detect the intracellular alkyl radical generation capacity of H + AB@Cu-MOF hydrogels. As shown in Figure 6i, almost no green fluorescence was observed in the control group or the pure H hydrogel group, indicating that the hydrogel itself could not trigger radical production. Only extremely faint fluorescence was detected in the non-irradiated H + AB@Cu-MOF group, suggesting that AIPH was hard to decompose and had trouble releasing alkyl radicals without an NIR-triggered photothermal effect. Only a small amount of ·OH produced from weak Cu-MOF-mediated Fenton reactions was insufficient to induce severe oxidative stress. The weak fluorescent signal of the H + A@Cu-MOF + NIR group proved that AIPH alone could hardly generate alkyl radicals without continuous photothermal support from ABTS·^+^. Moderate fluorescence enhancement was found in the Cu-MOF-free H + AB + NIR group, which was derived from NIR-induced AIPH decomposition. Nevertheless, the lack of GSH consumption and CDT synergy caused easy reduction and deactivation of ABTS·^+^ by GSH, thus leading to insufficient photothermal efficiency, limited alkyl radical output, and failure in ·OH production. By comparison, the intracellular green fluorescence intensity increased sharply in the H + AB@Cu-MOF + NIR group, which directly demonstrated that this system could induce massive alkyl radical burst via synergistic PTT-TDT effects and simultaneously produce abundant ·OH with the assistance of Cu-MOF, thus achieving massive intracellular radical accumulation. ROSup was set as the positive control to produce strong fluorescence and define the maximum response value of the detection system. These cellular experimental results verify the synergistic radical-producing effects of H + AB@Cu-MOF composite hydrogels based on PTT/TDT/CDT, which can induce severe oxidative stress damage and achieve efficient tumor cell ablation.

### 3.12. In Vivo Tumor Inhibition Effect of Nanocomposite Hydrogels

**Figure 7:**
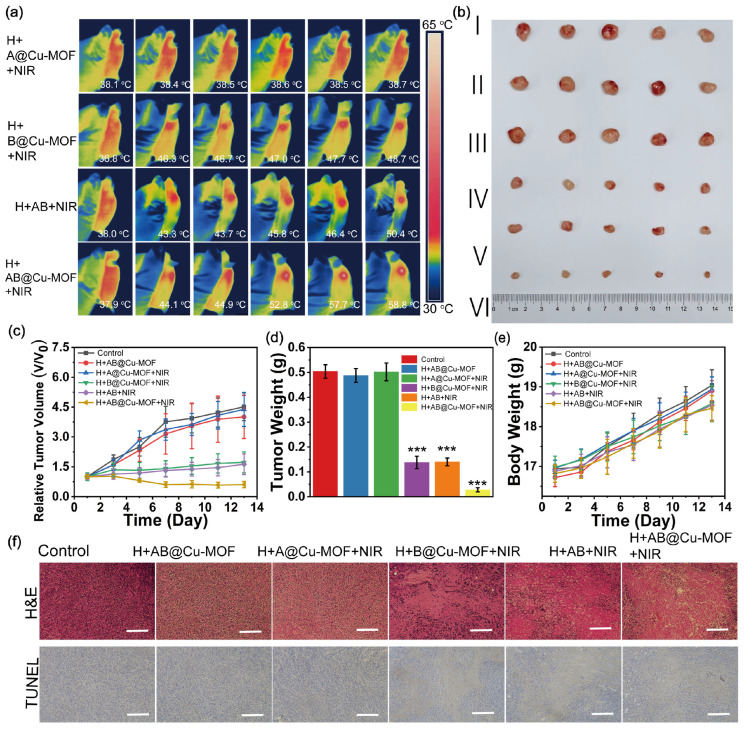
(**a**) In vivo infrared thermal images of mice in different treatment groups (H + A@Cu-MOF + NIR, H + B@Cu-MOF + NIR, H + AB + NIR, and H + AB@Cu-MOF + NIR). (**b**) Digital photographs of tumors from different groups on day 13: (**I**) PBS control group, (**II**) non-irradiated H + AB@Cu-MOF group, (**III**) H + A@Cu-MOF + NIR group, (**IV**) H + B@Cu-MOF + NIR group, (**V**) H + AB + NIR group, and (**VI**) H + AB@Cu-MOF + NIR group. (**c**) Average relative tumor volume during treatment (*n* = 5, mean ± SD). (**d**) Average tumor weight of each group on day 13 (*n* = 5, mean ± SD, *** *p* < 0.001). (**e**) Average body weight of mice during treatment (*n* = 5, mean ± SD). (**f**) H&E staining and TUNEL staining images of tumor tissues in different groups: PBS control group, non-irradiated H + AB@Cu-MOF group, H + A@Cu-MOF + NIR group, H + B@Cu-MOF + NIR group, H + AB + NIR group, and H + AB@Cu-MOF + NIR group (scale bar: 200 μm).

To evaluate the in vivo synergistic anti-tumor efficacy of NIR-irradiated H + AB@Cu-MOF hydrogels, subcutaneous pancreatic tumor models were established in nude mice. The mice were divided into groups and treated with PBS, non-irradiated H + AB@Cu-MOF, H + A@Cu-MOF + NIR, H + B@Cu-MOF + NIR, H + AB + NIR, and H + AB@Cu-MOF + NIR, respectively. All NIR irradiation was performed at 808 nm and 1 W/cm^2^ for 5 min each time on Days 1, 3, 5, 7, and 9 after treatment. As demonstrated by infrared thermal imaging in Figure 7a, the intratumoral temperature of the H + A@Cu-MOF + NIR group showed no obvious elevation, with a maximum temperature of only around 38.7 °C, indicating that single NIR irradiation exerted negligible photothermal effect without photothermal agents. In contrast, remarkable temperature elevation was observed in the H + B@Cu-MOF + NIR and H + AB + NIR groups, with maximum temperatures reaching 48.7 °C and 50.3 °C. Notably, the tumor temperature of the H + AB@Cu-MOF + NIR group kept rising and stabilized above 58.8 °C upon irradiation. This fully confirms that alkyl radicals derived from the AIPH and GSH scavenging capacity of Cu-MOF jointly boost the photothermal performance of ABTS·^+^, which is consistent with in vitro results and enables efficient local in vivo PTT. The in vivo photothermal effect can raise the local temperature to the T_VPT_ (43 °C) of the thermoresponsive hydrogel, triggering gel shrinkage and accelerated drug release. Although this temperature is close to the thermal damage threshold of normal tissues, the combined strategy of intratumoral administration and intermittent localized NIR irradiation adopted in this system fundamentally avoids non-specific thermal damage. After intratumoral injection, the hydrogel can stably reside at tumor sites and barely diffuse to surrounding tissues. Because NIR light only targets the lesion area, heat is strictly confined inside of tumors, keeping normal tissues at a safe temperature. Furthermore, replacing continuous irradiation with intermittent exposure enables precise regulation of peak temperature, which further guarantees thermal safety during repeated treatment cycles. Tumor volume monitoring results (Figure 7c) revealed that tumors in the PBS group grew rapidly and continuously, with the relative tumor volume increasing over 4.5 times within 14 days, confirming successful model establishment without spontaneous tumor regression. The relative tumor volumes of the non-irradiated H + AB@Cu-MOF group and the H + A@Cu-MOF + NIR group reached 4.0 times and 4.3 times the initial volume, respectively. These two groups only relied on Cu-MOF-mediated CDT to generate reactive oxygen species, thus merely mildly delaying tumor growth with limited therapeutic effects. The relative tumor volume of the H + B@Cu-MOF + NIR group decreased to 1.7 times owing to the combined effect of ABTS·^+^-based PTT and Cu-MOF-induced CDT via sustained hydroxyl radical production. Benefiting from the introduction of AIPH, the H + AB + NIR group achieved a relative tumor volume of 1.6 times, reflecting synergistic TDT and PTT effects. Nevertheless, its anti-tumor efficacy showed no distinct superiority over the single PTT group due to the absence of Cu-MOF-mediated GSH depletion and enhanced CDT. The H + AB@Cu-MOF + NIR combined treatment group exhibited the most prominent tumor suppression effect, with the relative tumor volume dropping to approximately 0.6 times the initial value within 14 days. This outstanding anti-tumor efficacy stems from the triple synergistic cascade of ABTS·^+^-based PTT, AIPH-mediated TDT, and Cu-MOF-catalyzed CDT. Cu-MOF pre-consumes intracellular GSH to preserve the photothermal activity of ABTS·^+^ and continuously generates cytotoxic ·OH to boost CDT performance. Upon NIR irradiation, heat triggers AIPH decomposition to yield alkyl radicals that drive TDT and simultaneously reinforce PTT. In addition, the thermoresponsive property of the hydrogel enables temperature-triggered controlled cargo release, which elevates local therapeutic concentrations and avoids systemic accumulation and accompanying side-effects on normal tissues. Tumor photographs and weight statistics on Day 13 after treatment (Figure 7b,d) further verified the above conclusions. The tumor size and weight of the H + AB@Cu-MOF + NIR group were significantly lower than those of all other groups with extremely significant differences compared with the control group (*** *p* < 0.001), which fully proves that AB@Cu-MOF nanocomposite hydrogels can achieve prominent synergistic enhancement of PTT-TDT-CDT under NIR irradiation and greatly improve in vivo anti-tumor efficacy. No obvious morphological abnormalities were observed in major organs, including the heart, liver, spleen, lung, and kidney, of mice in each group after treatment (Figure S7). Body weight monitoring results (Figure 7e) showed steady weight gain in all treatment groups without sharp declines or abnormal fluctuations during the 14-day observation period, preliminarily proving favorable in vivo biosafety of the prepared nanocomposite hydrogel system. H&E and TUNEL staining (Figure 7f) were adopted to systematically assess tumor tissue necrosis and cell apoptosis. Tumor cells in the PBS group presented regular morphology with intact nucleus and cell membrane structures without obvious pathological damage. By comparison, varying degrees of tissue damage were detected in all treatment groups, among which the H + AB@Cu-MOF + NIR group showed the most severe pathological changes, including extensive pyknosis, karyorrhexis, and karyolysis, with severely destroyed cell structures. These findings directly verify the potent anti-tumor activity realized by thermoresponsive controlled release of hydrogels combined with triple PTT-TDT-CDT synergistic therapy.

**Figure 8:**
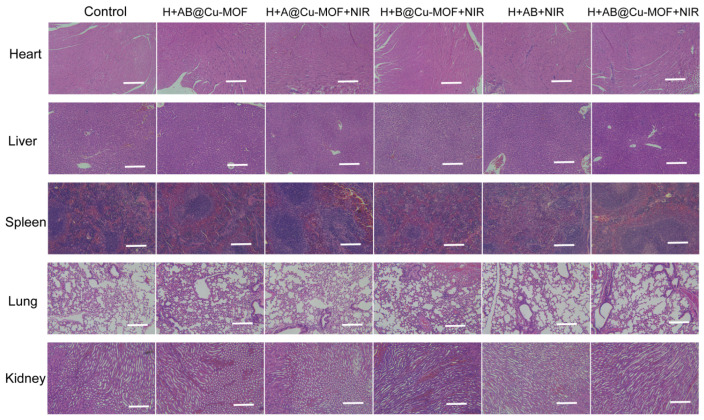
H&E staining images of the heart, liver, spleen, lung, and kidney tissues of mice in different treatment groups: PBS control group, non-irradiated H + AB@Cu-MOF group, H + A@Cu-MOF + NIR group, H + B@Cu-MOF + NIR group, H + AB + NIR group, and H + AB@Cu-MOF + NIR group (scale bar: 200 μm).

H&E staining was further used to evaluate the histopathological structures of major organs in mice of each group. The results showed that no obvious pathological damage, inflammatory infiltration, or necrotic lesions were observed in the heart, liver, spleen, lung, or kidney tissues of all treatment groups, which fully confirmed the excellent in vivo biosafety of this nanocomposite hydrogel system (Figure 8).

This work focuses on constructing a triple-modal synergistic therapeutic hydrogel system of PTT-TDT-CDT and elucidating its anti-tumor mechanism, which is a proof-of-concept study. The absence of positive control groups using mainstream chemotherapeutic drugs against pancreatic cancer such as gemcitabine is one of the limitations of this research. Currently, gemcitabine serves as the first-line chemotherapeutic agent for pancreatic ductal adenocarcinoma in clinical practice. It eliminates tumor cells via systemic administration. Nevertheless, the dense stromal barrier of pancreatic tumors greatly reduces drug penetration efficiency, and long-term administration tends to cause systemic side-effects and tumor drug resistance. In contrast, our system adopts local treatment via intratumoral injection. Tumor ablation is realized through the synergistic effects of the photothermal effect, alkyl radicals, and hydroxyl radicals. Without the application of conventional chemotherapeutic drugs throughout treatment, the system exhibits excellent biosafety and favorable local retention performance. These two therapies differ substantially in action mechanism, administration route, and application scenarios, and they are highly complementary to each other. In follow-up studies, we will add chemotherapeutic drug control groups and explore the combined application of this hydrogel system and clinical chemotherapy regimens so as to further improve the therapeutic efficacy evaluation system and promote clinical translation of the material.

Long-term biosafety is another major limitation of this study. Copper ions released from degraded Cu-MOF pose a risk of accumulation in the liver and kidney, and their in vivo distribution, biological fate, and metabolic pathways have not been verified via long-term animal experiments. Likewise, the long-term in vivo behaviors of AIPH, ABTS, and their degradation products remain to be tracked. In follow-up work, we will conduct long-term animal tests combined with ICP-MS, fluorescence tracing, pathological analysis, and other characterization techniques to systematically evaluate the metabolic profiles and long-term biosafety of each component.

## 4. Conclusions

Targeting the clinical challenges of extremely poor prognosis of PDAC, inoperable advanced cases, difficult delivery of chemotherapeutic drugs, and treatment resistance caused by the tumor microenvironment, this study focused on the limitations of low delivery efficiency and inadequate synergy in combined PTT and TDT strategies and constructed a multi-synergistic anti-tumor system based on injectable thermosensitive hydrogels. By dispersing Cu-MOF composites loaded with photothermal agent ABTS·^+^ and thermodynamic agent AIPH into APMOH/CMCS hydrogels, NIR-triggered self-accelerating circulation of PTT and TDT was realized. Combined with Cu-MOF-mediated CDT and the thermoresponsive release behavior of hydrogels, a triple synergistic therapeutic pattern of PTT-TDT-CDT was established. This system can penetrate tumor stromal barriers through intratumoral injection, achieve self-regeneration of photothermal agents via alkyl radicals, and simultaneously consume GSH and generate reactive oxygen species to boost therapeutic outcomes. It offers a new strategy and experimental foundation for breaking through the bottlenecks in pancreatic cancer treatment and achieving precise and efficient synergistic anti-tumor therapy. Furthermore, this integrated strategy of functional nanomaterials combined with intelligent hydrogels possesses good universality. The modular design can be extended to other solid tumors with similar tumor microenvironments, offering a valuable reference for the development of next-generation tumor therapeutic platforms.

## Data Availability

The original contributions presented in this study are included in the article/Supplementary Materials. Further inquiries can be directed to the corresponding author.

## Supplementary Materials

The following supporting information can be downloaded at https://www.mdpi.com/article/10.3390/polym18131620/s1, Text S1: Calculation of effect size; Figure S1: Full-range XPS spectrum of AB@Cu-MOF; Figure S2: FT-IR spectra of PMOH, APMOH, and APMOH/CMCS; Figure S3: GPC traces of PMOH and APMOH; Figure S4: Digital photographs showing volume shrinkage and hydrophilic–hydrophobic transition of APMOH/CMCS hydrogel loaded with AB@Cu-MOF under NIR irradiation; Figure S5: Absorbance changes at 734 nm reflecting ABTS·^+^ regeneration in GSH-treated AB@Cu-MOF with different concentrations of L-ascorbic acid (0.2 mg/mL, 0.2 mg/mL) under 808 nm of NIR irradiation (1 W/cm^2^); Figure S6: Three photothermal cycle curves and time constant τ fitting results of H + B@Cu-MOF, H + AB, and H + AB@Cu-MOF; Figure S7: Digital photographs of major organs (heart, liver, spleen, lung, kidney) from different treatment groups on day 13.
