# Supplementary material for: Thermoresponsive Injectable Self-Healing Hydrogel Loaded with Self-Regenerating Photothermal Agent for Synergistic Photothermal–Thermodynamic–Chemodynamic Therapy for Pancreatic Cancer
Source: Polymers (Basel). 2026 Jun 29;18(13):1620. doi: 10.3390/polym18131620 (PMC13364316; doi:10.3390/polym18131620)
Supplement: Supplementary file 1 [file polymers-18-01620-s001.zip › polymers-4374930-supplementary.pdf]

Supporting Information

Thermoresponsive injectable self-healing hydrogel loaded with self-regenerating photothermal agent for synergistic photothermal-thermodynamic-chemodynamic therapy of pancreatic cancer

Junhang Li[1], Weizhong Yuan[1*]

[1] School of Materials Science and Engineering, Tongji University, Shanghai 201804, P. R. China; 2331466@tongji.edu.cn (J.L.); yuanwz@tongji.edu.cn (W.Y.)

* Correspondence: yuanwz@tongji.edu.cn (W.Y.)

Text S1. Calculation of effect size

The effect size was calculated based on one-way ANOVA results of tumor weight. Between-groups sum of squares: $SS_{between}$; Total sum of squares: SS_{total}; Eta-squared: η^2; Cohen's effect size: f.

$$\eta^2 = \frac{SS_{between}}{SS_{total}} \text{ (S1)}, \quad \text{Cohen's } f = \sqrt{\frac{\eta^2}{1-\eta^2}} \text{ (S2)}$$

The one-way ANOVA results for tumor weight showed $SS_{between}$ = 1.2180 and SS_{total} = 1.2328. Calculations using Equations S1 and S2 yielded $\eta^2 \approx 0.9880$ and $f \approx 9.0700$. According to general statistical criteria, $f \geq 0.4$ indicates a large effect size. Such a prominent effect verifies that five mice per group is a reasonable sample size for detecting intergroup differences.

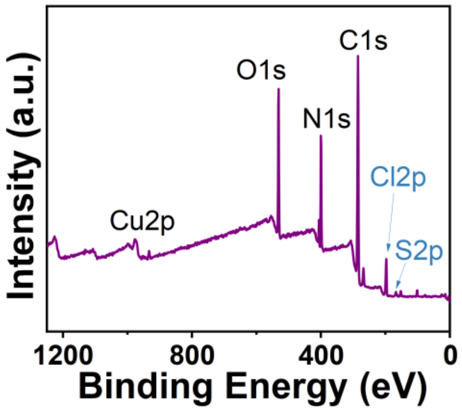

Figure S1. Full-range XPS spectrum of AB@Cu-MOF.

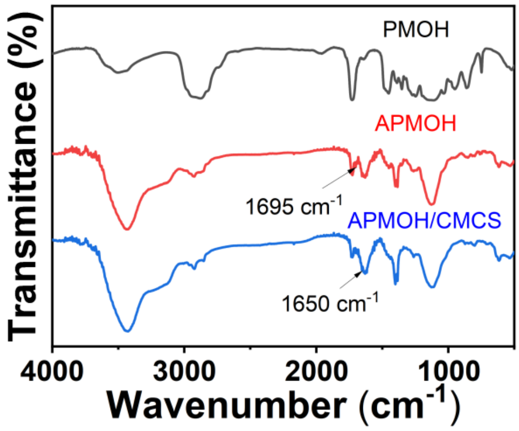

Figure S2. FT-IR spectra of PMOH, APMOH and APMOH/CMCS.

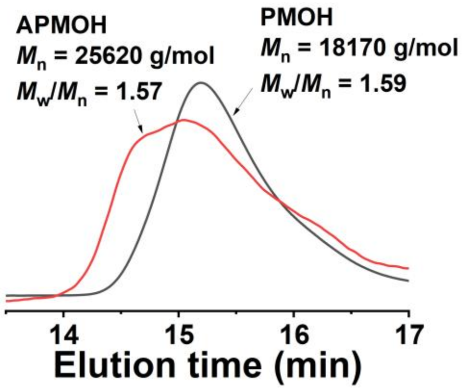

Figure S3. GPC traces of PMOH and APMOH.

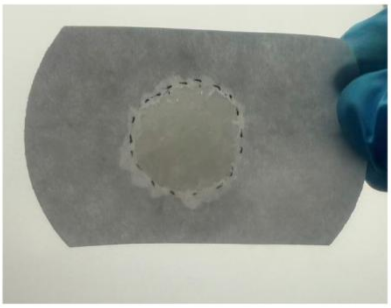

Figure S4. Digital photographs showing volume shrinkage and hydrophilic-hydrophobic transition of APMOH/CMCS hydrogel loaded with AB@Cu-MOF under NIR irradiation.

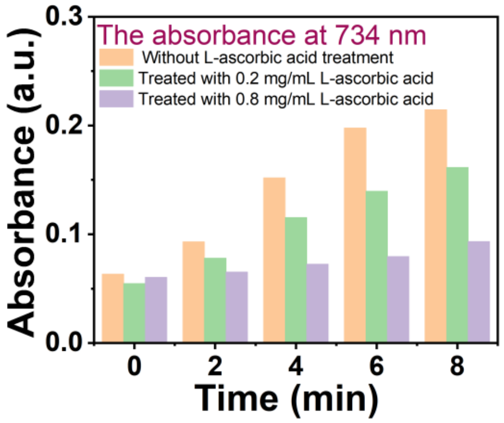

Figure S5. Absorbance changes at 734 nm reflecting ABTS$^{\cdot+}$ regeneration in GSH-treated AB@Cu-MOF with different concentrations of L-ascorbic acid (0.2 mg/mL, 0.2 mg/mL) under 808 nm NIR irradiation (1 W/cm^2).

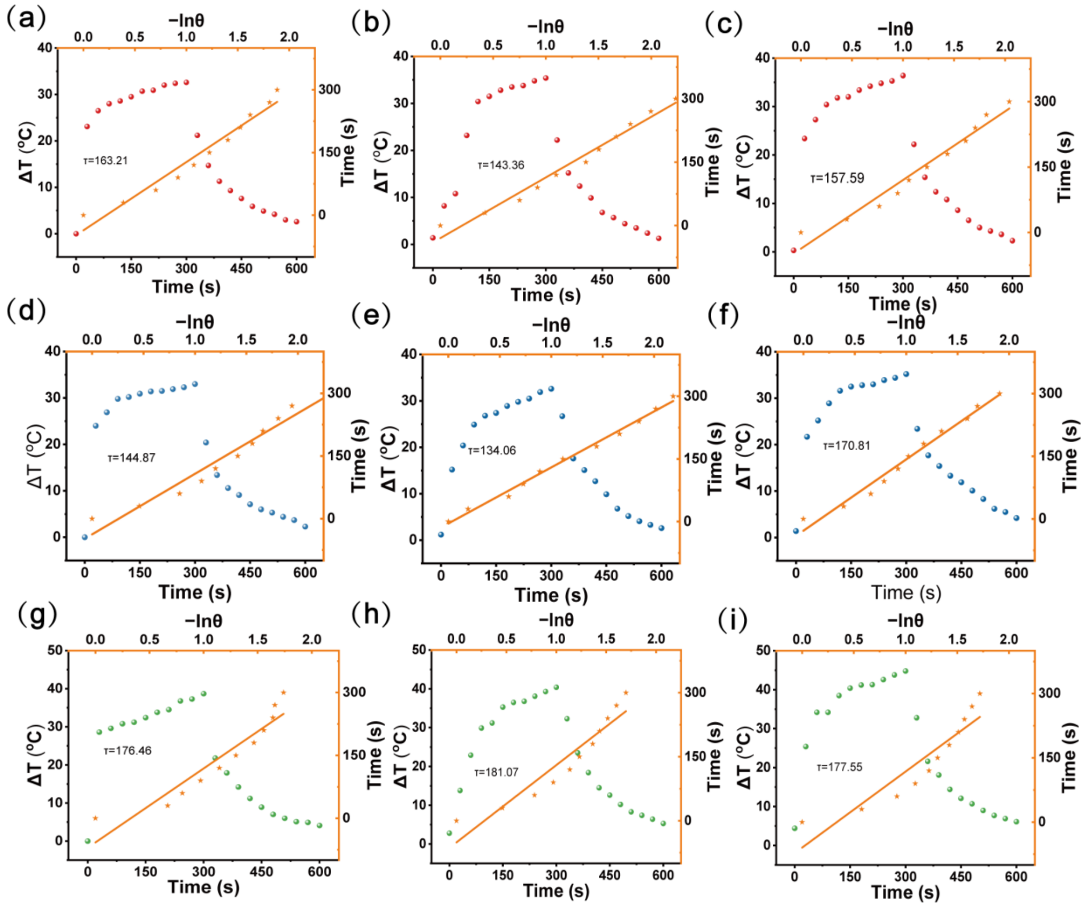

Figure S6. Three photothermal cycle curves and time constant τ fitting results of H+B@Cu-MOF, H+AB and H+AB@Cu-MOF.

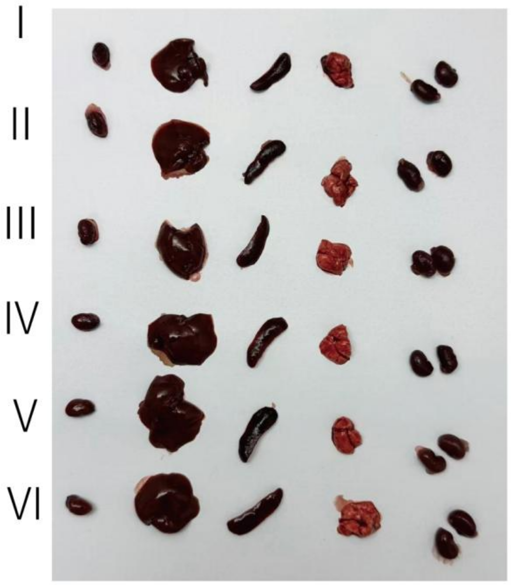

Figure S7. Digital photographs of major organs (heart, liver, spleen, lung, kidney) from different treatment groups on day 13.
